# Serum Thyroid Biomarkers for Diagnosing Malignant Thyroid Nodules: A Machine Learning Approach with External and Causal Validation

**DOI:** 10.7150/jca.131422

**Published:** 2026-05-18

**Authors:** Qingling Gu, Faling Xue, Xiaojing Shi, Zhuoling Zheng, Wenqing Wang, Zhuolin Yang, Yiyu Zhang, Wenqi Huang, Min Huang, Zhongxing Wang, Jiali Li

**Affiliations:** 1 School of Pharmaceutical Sciences, Sun Yat-sen University, Guangzhou, Guangdong, China.; 2 Department of Anaesthesiology, The First Affiliated Hospital of Sun Yat-sen University, Guangzhou, Guangdong, China.; 3 Department of Pharmacy, The Sixth Affiliated Hospital, Sun Yat-Sen University, Guangzhou, Guangdong, China.

**Keywords:** thyroid nodules, risk assessment, diagnostic biomarkers, machine learning, thyroglobulin

## Abstract

**Background:**

Thyroid nodules (TNs) are common, and accurate malignancy risk stratification is critical for clinical decision-making. Although serum thyroid biomarkers are routinely measured, their contribution to malignancy discrimination remains insufficiently characterized. Thus, we aimed to develop a practical and biologically grounded model for identifying malignant TNs using routinely clinical data.

**Methods:**

We retrospectively analyzed 5537 patients initially diagnosed with TNs from two hospitals, including 66.23% with malignant TNs. Diagnostic models were developed using logistic regression and machine learning approaches with internal and external validation. Causal associations were assessed using Mendelian randomization (MR), and biological relevance was further examined based on TCGA data.

**Results:**

Younger age (OR = 0.96 [0.95-0.97], P < 0.001) and lower thyroglobulin (Tg) levels (OR = 0.64 [0.61-0.67], P < 0.001) were identified as key risk factors for malignant TNs. The two-factor model achieved AUCs of 0.755 (training), 0.735 (internal validation), 0.76 (temporal validation), and 0.784 (external validation) among various cohorts, and demonstrated incremental value to TI-RADS. An interactive web-based nomogram was developed as a proof-of-concept tool to support clinical utility. MR analysis supported a positive causal relationship between Tg and benign TNs (β = 0.62, P = 0.006), while TCGA data confirmed the inverse association between Tg and malignant TNs (OR = 0.14, P < 0.001).

**Conclusion:**

A simple model integrating age and routine serum Tg demonstrates robust and generalizable performance for malignancy risk stratification of TNs. By integrating clinical prediction with causal inference and biological validation, this study provides a complementary tool to existing ultrasound-based risk stratification systems.

## Introduction

Thyroid nodules (TNs) are common clinical findings. Although most TNs are benign and pose minimal health risks to patients, approximately 10%-15% are found to be malignant—a proportion that has been steadily increasing in recent years 1,2. Malignant TNs often require total thyroidectomy and, in some cases, cervical lymph node dissection, underscoring the clinical importance of distinguishing malignant from benign nodules to prevent overtreatment 1-3. Ultrasonography, such as the American College of Radiology Thyroid Imaging Report and Data System (ACR TI-RADS), is the primary tool for TNs, while fine-needle aspiration (FNA) serves as a further diagnostic step. However, FNA is typically recommended only for nodules larger than 1-2 cm and carries a risk of false-negative results 4. Therefore, for patients requiring further evaluation, a comprehensive approach to supplement the TI-RADS is particularly crucial.

TNs can lead to various thyroid dysfunction 5-7, which is typically assessed by measuring serum levels of thyroid-stimulating hormone (TSH), triiodothyronine (T3), and thyroxine (T4). TSH stimulates the production of T3 and T4, which, once secreted into the bloodstream, are either bound to plasma proteins for storage or circulate in free form (FT3, FT4) to exert biological effects. Previous studies have reported that elevated TSH levels may be associated with an increased risk of thyroid cancer 8,9, while alterations in T3 and T4 levels may reflect the metabolic activity and proliferative status of thyroid tissues 10,11. In addition, several thyroid biomarkers, including thyroglobulin (Tg), a substrate for T3 and T4 synthesis, anti-thyroglobulin antibody (TGAB) and thyroid peroxidase antibody (TPOAb), important autoimmune markers, have been implicated in various thyroid diseases 12.

Traditionally, basic information such as age and sex has been recognised as important predictors of malignant TNs 1,2. However, there is still no clear consensus regarding the diagnostic value of thyroid biomarkers in relation to malignant TNs risk—particularly in the Chinese population. Given the routine use of thyroid function tests in clinical practice, investigating the diagnostic value of thyroid hormones and autoantibodies for the increasingly prevalent TNs holds substantial clinical relevance. Therefore, this study aims to clarify the association between TNs and thyroid biomarkers, and to construct a non-invasive diagnostic model as a simple adjunct to TI-RADS for malignancy risk assessment, so as to support more informed decisions regarding further evaluation (e.g., FNA) without additional procedural burden.

## Materials and Methods

### Clinical Data Collection

This retrospective study was conducted in accordance with the STROBE guidelines. Patients aged ≥18 years who were initially diagnosed with TNs were retrospectively recruited from the First Affiliated Hospital of Sun Yat-sen University between September 2014 and December 2023, and from the Sixth Affiliated Hospital of Sun Yat-sen University between January and December 2024. The study was approved by the Ethics Committees of both hospitals (Approval Number: [2024]772 and 2025ZSLYEC-328), with a waiver of informed consent granted. Patients with missing data or a history of taking thyroid hormone modulators within three months were excluded.

**Figure 1:**
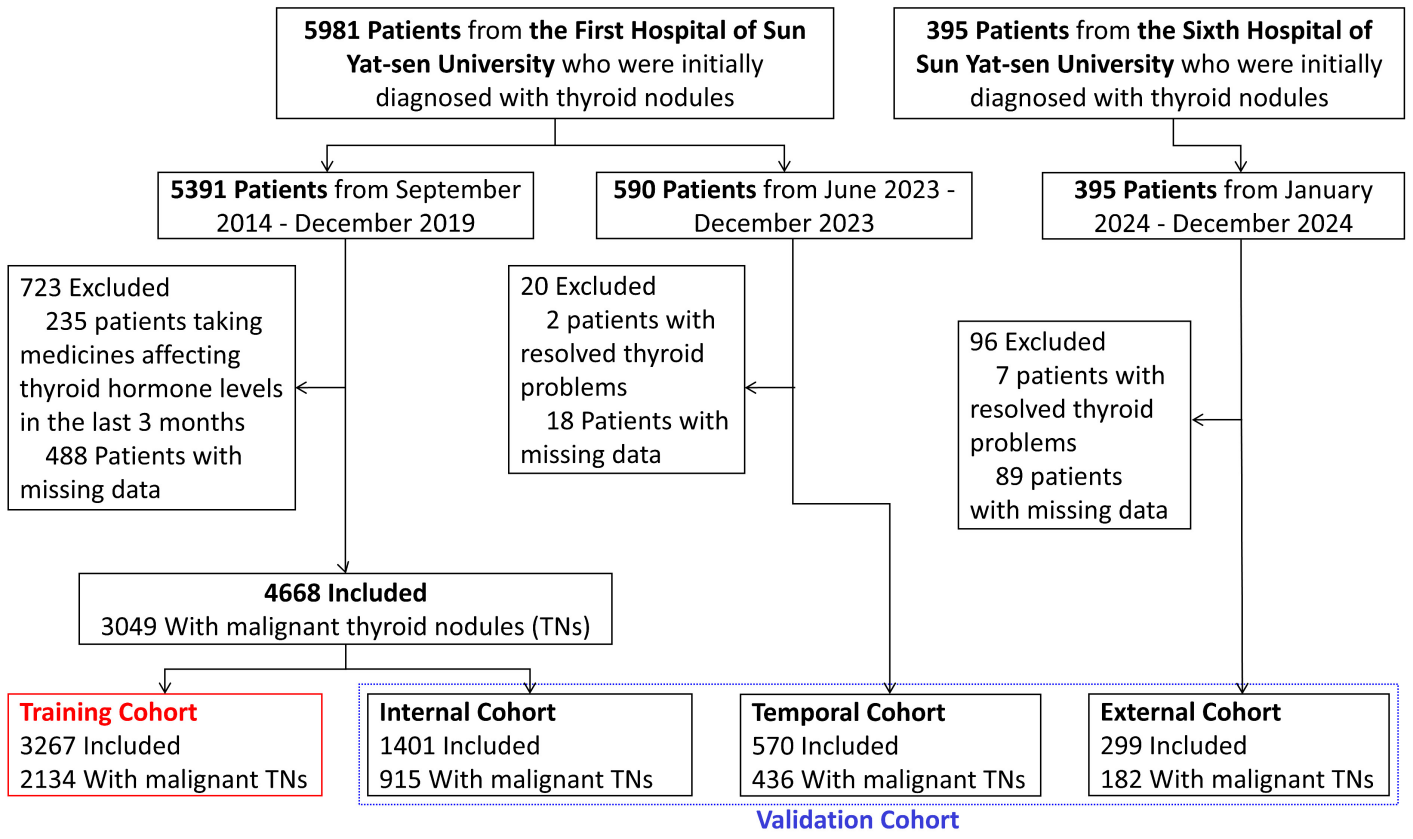
Flowchart of patient selection and dataset allocation.

An independent prospective cohort from the First Affiliated Hospital of Sun Yat-sen University was included for external temporal validation (Approval Number: [2014]141). This cohort was originally established for purposes unrelated to TNs malignancy prediction but contained relevant demographic information, serum Tg measurements, and definitive pathological outcomes. The Tg-age model was not retrained on this dataset and was evaluated exclusively for independent performance assessment. The primary outcome was nodule malignancy status, which was determined based on postoperative pathological examination. At last, a total of 5537 patients were included in the final analysis (Figure 1).

### Feature Screening by Machine Learning

We employed both logistic regression (LR) and various machine learning algorithms—including LASSO regression, Random Forest (RF), Gradient Boosting Decision Tree (GBDT), Extreme Boost Gradient Boosting (XGBoost), and Support Vector Machine (SVM)—to select important features for malignant TNs. Feature selection was performed using two strategies: one included all available data, while the other was limited to demographic characteristics and thyroid biomarkers. To ensure the feasibility of machine learning analysis, we performed median imputation for variables with <20% missing data. Variables with more than 20% missing data were excluded from the analysis. Supplementary Table 1 details the sample sizes of all candidate variables. It should be noted that there were no missing values for age or serum thyroid biomarkers (TSH, Tg, et al.) in our study population.

The dataset was randomly split into a training set (70%) and a test set (30%) for model development and validation (Figure 1). All machine learning models underwent repeated cross-validation for optimal performance tuning. SHapley Additive exPlanations (SHAP) values were calculated to determine the relative importance of each variable. A clinical variable with a positive SHAP value indicated an increased likelihood of malignancy, whereas a negative SHAP value suggested a reduced risk. Model discrimination ability was assessed using the area under the receiver operating characteristic curve (AUC).

### Model Construction and External Validation

Based on the selected features, we developed the LR model through backward stepwise selection method. Model performance during variable selection was assessed using multiple complementary metrics, including the Akaike Information Criterion (AIC), AUC, DeLong's test, and integrated discrimination improvement (IDI). Model robustness was evaluated through internal and external validation. The temporal validation cohort comprised 134 benign and 436 malignant TN cases recruited from the First Affiliated Hospital of Sun Yat-sen University between June and December 2023, while the external validation cohort included 117 benign and 182 malignant TN cases from the Sixth Affiliated Hospital of Sun Yat-sen University between January and December 2024 (Figure 1). In addition, stratified analyses were performed according to sex, serum TSH levels, and other clinically relevant variables to assess model stability across subgroups.

### Ultrasound Data Extraction and Analysis

To evaluate the incremental value of the Tg-age model beyond ultrasound-based risk stratification, we collected available TI-RADS grade and nodule size data from the external validation cohort. Finally, 165 patients with complete thyroid ultrasound reports were included. Where a patient had multiple nodules, the nodule with the highest TI-RADS grade was selected for analysis; where grades were identical, the largest nodule was chosen as the index nodule. Nodule size was recorded as the maximum diameter (mm) measured along three orthogonal axes. Diagnostic performance of TI-RADS alone, the Tg-age model alone, and their combination was assessed by receiver operating characteristic (ROC) curve analysis. Subgroup analysis was performed in patients with TI-RADS 3-4 nodules to assess the model's utility in indeterminate nodules.

### Web-based Dynamic Nomogram Deployment

To illustrate potential clinical applicability, we developed an interactive web-based nomogram and deployed it as an open-access application through the shiny platform (https://guqling.shinyapps.io/ThyroidNoduleDiagnosis-InputAge-lnTg-ngmL/). The tool allows users to input patient-specific variables (age and serum Tg levels) to generate individualized malignancy risk estimates with corresponding confidence intervals. The application is intended as a proof-of-concept demonstration rather than for direct clinical use.

### Causal Association Verification based on Mendelian randomization

We conducted two-sample Mendelian Randomization (MR) analyses to evaluate the causal association between thyroid biomarkers and TNs with the inverse-variance weighted (IVW) method as primary approach 13. Genome-Wide Association Study (GWAS) data for TNs were obtained from the latest FinnGen study, comprising 180928 individuals for benign TNs (CD2_BENIGN_THYROID_EXALLC) and 174995 for malignant TNs (C3_THYROID_GLAND_EXALLC). In both datasets, controls were cancer-free individuals. GWAS data for Tg were retrieved from the IEU OpenGWAS database (prot-a-2960). Summary statistics for TSH, FT3, FT4, TT3, and TPOAb were extracted from the ThyroidOmics Consortium, the largest GWAS of thyroid function. TT4 data were obtained from the GWAS Catalog (GCST90012759) 14. TGAB data were derived from a GWAS research 15.

### Biological Rationale Verification based on the TCGA Database

We validated the biological rationale for Tg as the key factor using data from the Cancer Genome Atlas (TCGA). First, we compared TG expression levels between thyroid tumor tissues and adjacent normal tissues, followed by Kaplan-Meier analysis to evaluate the association between TG and disease-free survival. Then, we performed differential gene expression (DGEs) analysis between samples with higher TG expression (top 10%) and lower expression (bottom 10%). Functional enrichment analysis, including Gene Ontology (GO), Kyoto Encyclopedia of Genes and Genomes (KEGG), and Gene Set Enrichment Analysis (GSEA), was also performed based the MSigDB database. Lastly, we applied the CIBERSORT-ABS algorithm to assess the relationship between TG expression and immune cell infiltration, which is optimized for cross-sample comparison of immune cell fractions 16.

### Statistical Analysis

Continuous variables were presented as median and interquartile range (IQR) and categorical variables were expressed as counts (percentages). For variables with highly skewed distributions, we applied natural logarithm (ln) transformation or categorized them based on clinically defined reference ranges. Univariate LR was used to assess the association between each variable and the outcome. The key factors were screened by various feature selection methods and based on this, a diagnostic model for malignant TNs was developed by multivariable LR. A p-value < 0.05 or false discovery rate (FDR) < 0.05 indicates statistically significant.

All statistical analyses were performed using R (version 4.3.2). The following R packages were used: gtsummary (v2.0.0), rms (v6.7-1), pROC (v1.18.5), and car (v3.1-2) for data analysis; randomForest (v4.7-1.1), xgboost (v1.7.8.1), e1071 (v1.7-14), and caret (v6.0-94) for machine learning; rsconnect (v1.4.1), shiny (v1.10.0), and DynNom (v5.1) for web tool deployment; TwoSampleMR (v0.6.3) and ieugwasr (v1.0.1) for MR analysis; DESeq2 (v1.42.1), clusterProfiler (v4.12.0), and org.Hs.eg.db (v3.18.0) for transcriptome analysis; ggplot2 (v3.5.1), forestplot (v3.1.3), EnhancedVolcano (v1.24.0), and enrichplot (v1.22.0) for visualization.

## Results

### Sample Characteristics in Our Cohorts

A total of 5537 patients were included, with a median age of 43 years. Among them, 3667 participants had malignant TNs (Figure 1). Table 1 summarizes the demographic characteristics and thyroid biomarkers of the overall cohort and subgroup cohorts. There was no significant difference in sex distribution. However, patients with malignant TNs were significantly younger. Most thyroid biomarkers differed between subgroups, with malignant TNs exhibited lower total triiodothyronine (TT3) and Tg levels, and higher TSH, TGAb, and TPOAb levels. We also observed that patients with malignant TNs had a longer postoperative hospital stay and higher medical costs. Nonetheless, even patients with benign TNs incurred a median cost of ¥15387, posing a substantial burden on both families and the healthcare system. Therefore, effectively distinguishing benign TNs to avoid overtreatment remains a pressing medical challenge.

### Association Between Candidate Variables and Malignancy Risk

A total of 75 routinely measured blood biomarkers, including thyroid-related indices, were collected at the initial clinical evaluation. Univariate LR analysis revealed that age, TSH, and Tg levels were significantly associated with malignant TNs (P < 0.001, AUC > 0.6) (Supplemental Table 1). Specifically, younger age (OR = 0.96, 95% CI: 0.95-0.96; P < 0.001), higher serum TSH levels (OR = 1.21, 95% CI: 1.15-1.27; P < 0.001), and lower Tg levels (lnTg; OR = 0.64, 95% CI: 0.61-0.66; P < 0.001) were independently associated with malignant TNs. Further analysis indicated that Tg could effectively differentiate between benign and malignant TNs at a cutoff value of 24.72 ng/mL (AUC = 0.722) (Supplemental Figure 1). Although the conventional normal range for Tg is 1-40 ng/mL, introducing a subclinical threshold of 24.72 ng/mL may provide valuable diagnostic utility in the early detection of malignancy.

### Model Construction and External Validation

Baseline characteristics and outcomes of the training set (n = 3394, 70%) and test set (n = 1401, 30%) are summarized in Supplemental Table 2, with no significant differences observed between the two sets. The discrimination performance of different models during feature screening is presented in Supplemental Table 3. Including the full panel of available variables did not materially improve the model discrimination, and the performance of LR, LASSO, GBDT and XGBoost remained comparable when the model was restricted to only demographic characteristics and thyroid biomarkers. Given that interpretability and practicability are as important as predictive performance, we selected the LR model for subsequent analyses, as tree-based models, although robust, remain more complex to interpret despite the application of SHAP analyses (Supplemental Figures 2 and 3).

We selected only demographic characteristics and thyroid biomarkers to construct the final diagnostic model. Table 2 summarizes the stepwise logistic regression process across the training, internal validation, and external validation cohorts. Although multiple thyroid biomarkers were significantly associated with nodule malignancy, age and Tg emerged as the most robust independent factors, in line with the results of XGBoost and GBDT. Younger patients and those with lower Tg levels were significantly more likely to have malignant TNs. Combined, these two variables could distinguish benign from malignant TNs with an AUC of 0.755. Compared with the full variable model, the performance of the stepwise model showed slight reduction in both the internal validation set (IDI = -0.008) and the external validation set (IDI = -0.002). Baseline characteristics and outcome comparisons for the external validation cohort are detailed in Supplemental Table 4.

To further assess model generalizability, we evaluated the Tg-age model in an independent prospective cohort from the First Affiliated Hospital of Sun Yat-sen University, which was originally established for purposes other than TN malignancy prediction. This cohort included 134 benign and 436 malignant TN cases (Supplemental Figure 4). The model achieved an AUC of 0.76 (95% CI: 0.708-0.812), consistent with its performance across other validation cohorts. These results support the robustness and temporal generalizability of the model across different populations and data collection settings. Finally, a dynamic web-based nomogram was developed based on the final two-factor model to enable interactive prediction of malignancy risk in patients with TNs. The application allows users to input basic clinical parameters and obtain individualized risk estimates in real time.

### Integration with Ultrasound-based Risk Stratification

**Figure 2:**
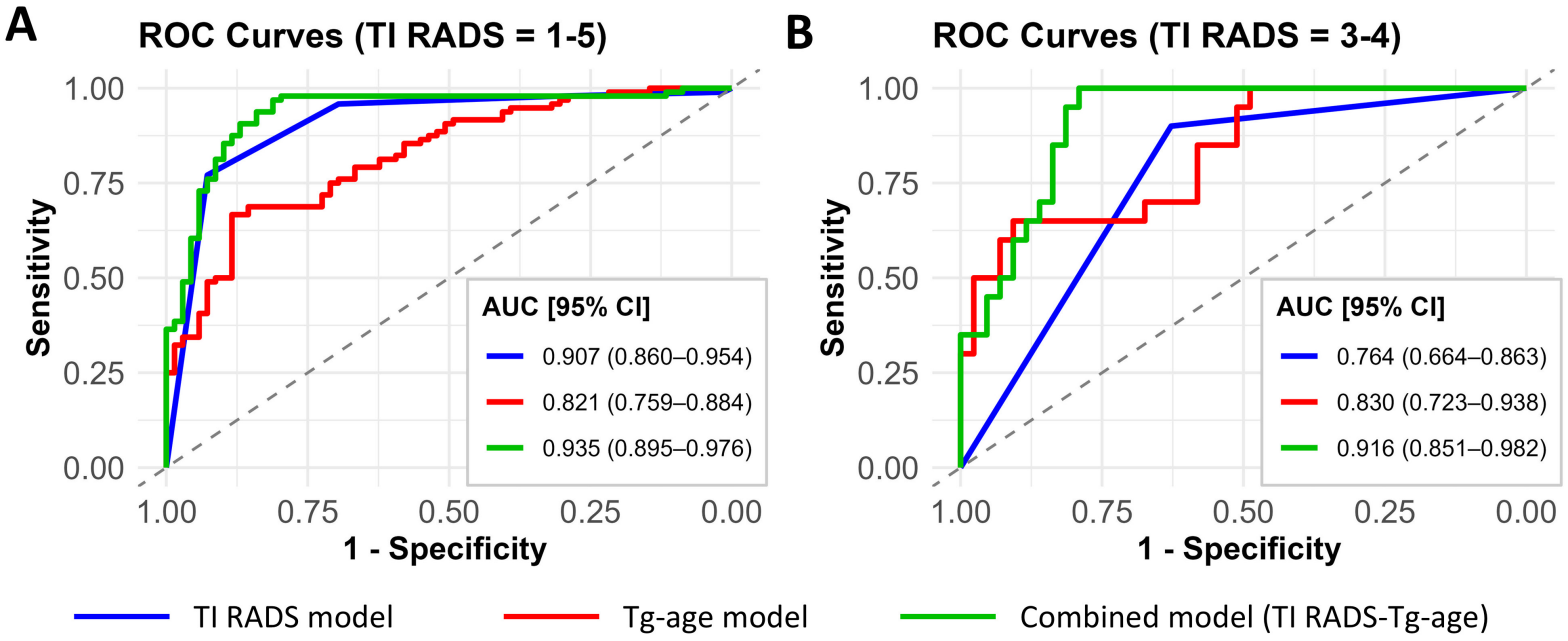
** Receiver Operating Characteristic (ROC) Curves of Different Models for Malignancy Prediction in Thyroid Nodules.** A. The performance in all patients with available TI-RADS data (n = 165). B. The performance in patients with indeterminate nodules (TI-RADS = 3-4) (n = 78). The area under the curve (AUC) with 95% confidence intervals for each model is presented. The diagonal dashed line represents the reference line (AUC = 0.5).

To evaluate the incremental value of the Tg-age model beyond ultrasound-based risk stratification, we compared the diagnostic performance of TI-RADS alone, the Tg-age model alone, and their combination in the external validation cohort (Supplemental Table 5). As shown in Figure 2, the TI-RADS model alone achieved an AUC of 0.907 (95% CI: 0.867-0.947). When combined with Tg-age, the AUC improved to 0.935 (95% CI: 0.902-0.968), indicating enhanced discriminative ability. Notably, in the subgroup of patients with TI-RADS 3-4 nodules, the TI-RADS model alone showed limited performance (AUC 0.764 [95% CI: 0.672-0.856]), whereas the combined model yielded a substantially improved AUC of 0.916 (95% CI: 0.857-0.975). These findings suggest that the Tg-age model serves as a valuable complement to TI-RADS, particularly for refining risk stratification of indeterminate nodules.

Given that larger benign nodules may produce more Tg. We also conducted a sensitivity analysis of nodule size. We found the coefficient of Tg remains statistically significant after adjusting for size (Supplemental Table 6), which suggests that, while nodule volume does influence Tg production, the negative correlation between malignancy and Tg secretion is not merely a byproduct of nodule size. Furthermore, we further integrated nodule size data into the combined model (TI-RADS = 1-5). Adjusting for nodule size had only a minor impact on model performance (AUC = 0.939 [95% CI: 0.901-0.976]), indicating that most nodule size information is already captured within the clinical context, and the incremental value of our model lies in its unique biological perspective.

### Diagnostic Performance Across Subgroups

To assess model robustness, stratified analyses were performed across subgroups defined by sex, TSH, TGAb, and TPOAb levels (Supplemental Table 7). The Tg-age model maintained stable discrimination across all subgroups in both internal and external validation cohorts, supporting its generalizability. Notably, model performance was consistently higher in the low-TSH subgroup (defined by the median value, TSH ≤ 1.5 mIU/L) than in the high-TSH subgroup across all cohorts, with an AUC of 0.792 (95% CI: 0.727-0.858) in the external validation cohort.

To further elucidate the influence of TSH levels on the Tg-age model, additional subgroup analyses were conducted under specific clinical contexts. In patients with Hashimoto's thyroiditis, the Tg-age model achieved an AUC of 0.700 (95% CI: 0.595-0.805). In the subclinical hypothyroidism subgroup, the Tg-age model achieved an AUC of 0.741 (95% CI: 0.623-0.859), whereas in the subclinical hyperthyroidism subgroup, the AUC decreased to 0.684 (95% CI: 0.593-0.776). In patients with hyperthyroidism, thyroid follicles are in a state of hypermetabolism, and serum Tg levels are elevated regardless of the underlying nature, which may mask the contribution of nodule-derived Tg, thereby reducing the model's ability.

### Multiple Validation of the Differentiation Capability for Thyroglobulin

**Figure 3:**
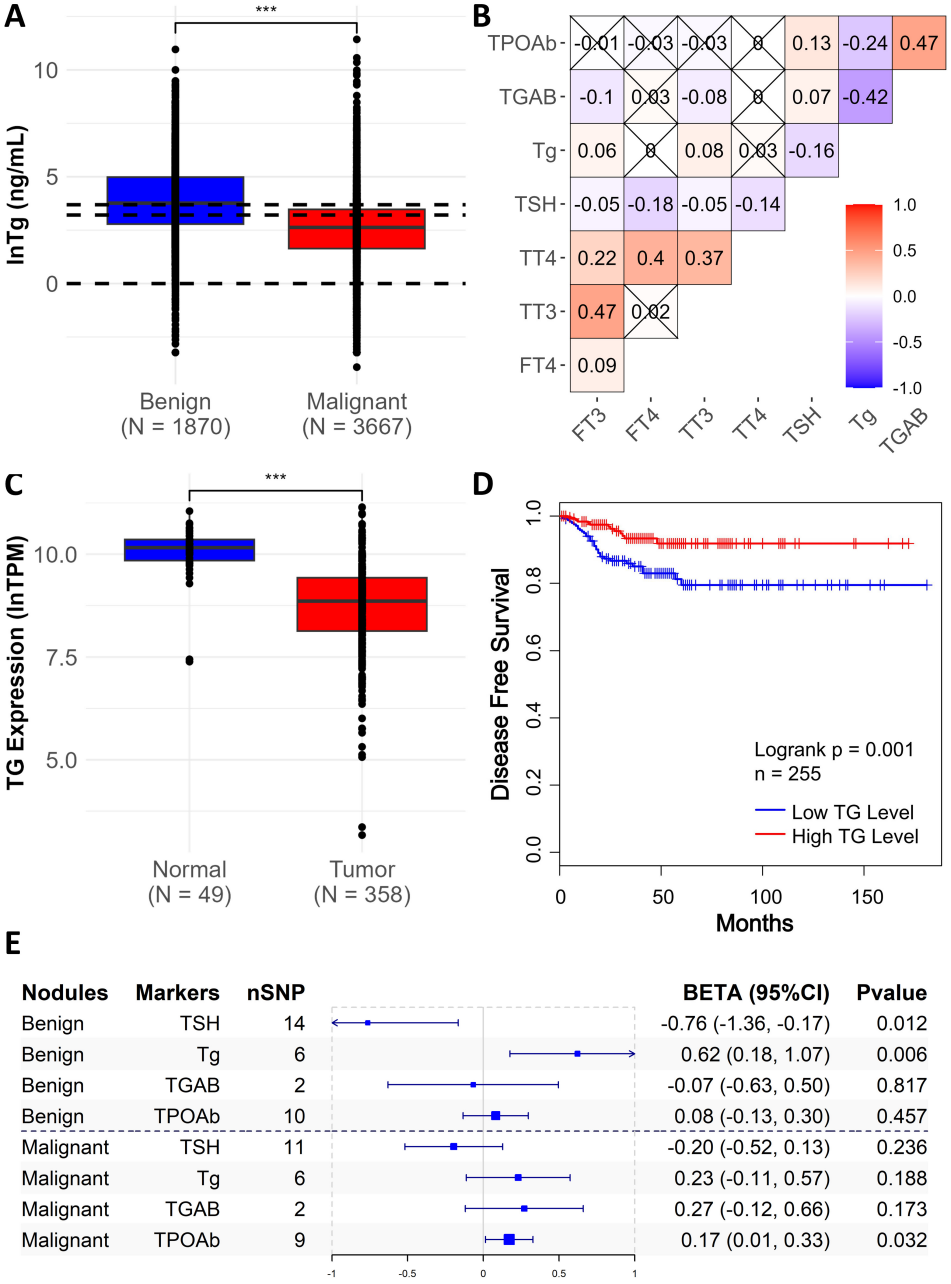
** Multiple evidence supporting thyroglobulin as a key biomarker of malignant thyroid nodules.** A. The distribution of thyroglobulin (Tg) across benign and malignant nodules. The black dashed lines show Tg = 1, 24.72, and 40 ng/mL. The statistical significance computed by the Wilcoxon test is annotated by the number of stars (***: P value <0.001). B. Correlation heatmap of thyroid-related biomarkers. The degree of correlation between different thyroid biomarkers is represented by color intensity. The symbol "×" indicates a P value > 0.05 after false discovery rate correction by Benjamini-Hochberg method. C. The gene expression distribution of thyroglobulin (Tg) in adjacent normal tissues and tumor tissues in the TCGA cohort. TPM, transcripts per million. D. Kaplan-Meier survival analysis of TG gene expression and disease free survival in TCGA cohort. E. Mendelian randomization analysis showed the causal association between thyroid biomarkers and thyroid nodules. nSNP indicates the number of instrumental single nucleotide polymorphisms used; BETA and 95% confidence intervals represents the estimated causal effect, while BETA > 0 indicates a positive causal association.

Evidence from multiple independent datasets consistently supports serum Tg as a key biomarker of malignant TNs. In our clinical cohort, lower Tg levels were strongly associated with malignancy: 79.5 % of patients with Tg < 24.72 ng/mL had malignant TNs (Figure 3A). Correlation analysis of thyroid biomarkers revealed that Tg was positively correlated with FT3 and TT3, and inversely correlated with TSH, TGAb, and TPOAb (Figure 3B), which means the lower the TSH, the higher the Tg. This pattern is biologically consistent with Tg reflecting preserved thyroid differentiation and hormone synthesis, whereas elevated TSH and thyroid autoantibodies are commonly associated with impaired thyroid function or autoimmune activity. These distinct correlation patterns support the biological relevance of Tg as an indicator of thyroid functional integrity in the context of TN malignancy.

Transcriptomic data from TCGA further reinforced the biological relevance of Tg. Compared with adjacent normal tissue, tumor samples showed significantly lower TG mRNA expression (OR = 0.14 [0.081, 0.243], P < 0.001) (Figure 3C), and higher TG expression was associated with favorable disease-free survival (Figure 3D). We also verified the causal association between Tg and TNs through MR analysis and found that Tg levels were significantly positively correlated with benign TNs (BETA = 0.62, P = 0.006), further verifying the differentiation capability of Tg (Figure 3E). MR analyses also indicated a negative association between TSH and benign TNs (β = -0.76, P = 0.012) and a positive association between TPOAb and malignant TNs (β = 0.17, P = 0.032), mirroring trends observed in our clinical cohort, equally validating the plausibility of our findings. Instrumental single nucleotide polymorphisms used in the MR analyses are listed in Supplemental Table 8, and full MR results are provided in Supplemental Table 9.

### Biological Functional Exploration of Thyroglobulin in Thyroid Nodules

**Figure 4:**
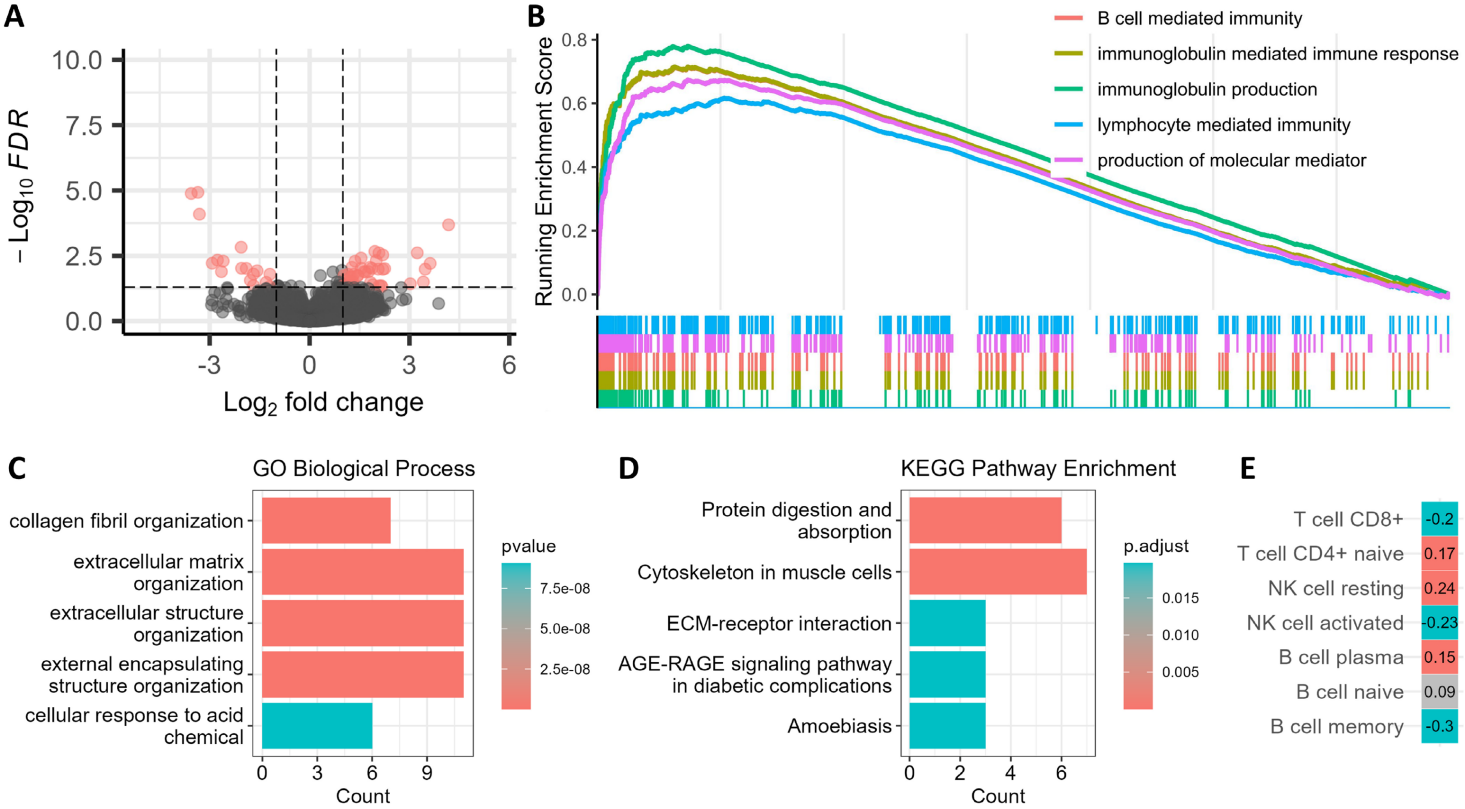
** Exploration of the potential biological functions of thyroglobulin in thyroid nodules.** A. Volcano plot showing differentially expressed genes (DEGs) between high and low TG expression groups with a threshold of |log₂ Fold Change| > 1 and a false discovery rate (FDR) < 0.05. B. Gene Set Enrichment Analysis (GSEA) based on Hallmark gene sets from MSigDB. C. Gene Ontology (GO) enrichment analysis of DEGs in the Biological Process category. D. Kyoto Encyclopedia of Genes and Genomes (KEGG) pathway enrichment analysis of DEGs. E. Correlation analysis between TG expression and immune cell infiltration.

To elucidate the biological plausibility of Tg as a key biomarker, we performed transcriptomic analyses based on TCGA data. DGEs and GSEA analysis showed that high TG expression was significantly enriched in pathways related to immunoglobulin production and immune responses, suggesting that Tg may be closely related to immune microenvironment (Figure 4A, B). GO analysis revealed enrichment in collagen fiber remodeling and extracellular matrix (ECM) organization, while KEGG pathway analysis indicated associations with the protein digestion and absorption and ECM-receptor interaction (Figure 4C, D). These pathways are known to mediate interactions between extracellular molecules and cell surface receptors, influencing intracellular signaling processes, highlighting a potential role for Tg in tumor microenvironment regulation.

To further investigate the link between Tg and immune microenvironment, we applied CIBERSORT-ABS to estimate immune cell infiltration across samples (Figure 4E). We found TG expression was positively correlated with CD4⁺ naive T cells, resting NK cells, and B cells, which are often related to unactivated immune environments in non-malignant tissues. In contrast, TG expression was negatively correlated with CD8⁺ T cells, activated NK cells, and memory B cells, typically associated with immune evasion in malignant tumor tissues. All these results indicate that TG expression can reflect the differences of immune microenvironment, and thereby reflect the malignancy of TNs. Cross-validation by additional methods like TIMER and xCell confirmed the consistency of these immune infiltration patterns (Supplemental Table 10).

## Discussion

Based on a multi-centre, large-scale cohort, the study constructed and validated a parsimonious two-factor model for identifying malignant from benign TNs. The final model relies solely on age and serum Tg levels, yet demonstrates robust diagnostic performance. It achieved an AUC of 0.755 in the training cohort, 0.735 in the internal validation cohort, 0.76 in the temporal validation cohort, and 0.765 in the external validation cohort. The high accuracy and generalisation ability of this model highlight its potential as a low-cost and efficient diagnostic tool for clinical application, so as to supplement existing ultrasound-based risk stratification systems.

There are various factors that influence the incidence of malignant TNs. Consistent with current guidelines 1, our cohort showed that males, younger age, high TSH and TPOAb levels were risk factors. Moreover, we confirmed the causal link between lower TSH and benign TNs, higher TPOAb and malignant TNs. Although TNs are more prevalent in females, our results support the established finding that males are more likely to develop malignancy in TNs carriers, potentially due to sex hormone-mediated mechanisms 17. In addition, we observed that even within the normal range, elevated TSH were associated with increased risk of malignancy 18,19, likely reflecting its pro-proliferative effect 20. Meanwhile, higher TPOAb were more frequently found in patients with malignant TNs. As a marker of thyroid autoimmunity 21, elevated TPOAb reflects an ongoing chronic inflammatory state, which may contribute to cellular damage and carcinogenesis 22.

Following stepwise regression, only serum Tg remained as an independent biomarker. Adding age improved predictive stability while maintaining model simplicity. Although postoperative Tg is well established for monitoring recurrence or metastasis in differentiated thyroid cancer after total thyroidectomy 23,24, its role as a preoperative biomarker for malignancy risk stratification remains controversial 25,26. Uysal S et al. 27 found that low Tg levels in FNA washings (FNA-Tg) may indicate malignant nodules, with significantly higher FNA-Tg in benign nodules (p < 0.001). When Midkine (a heparin-binding growth factor) was introduced as a reference, the Midkine/Tg ratio demonstrated good diagnostic performance for thyroid carcinoma 28,29. However, some studies found no predictive value of preoperative Tg for malignancy 30, and others even propose that elevated preoperative plasma Tg 31 or elevated FNA-Tg 32 could serve as markers for malignant nodules. Overall, these studies predominantly consist of small-scale, single-centre studies initiated by European research groups, their conclusions therefore have limited applicability.

Our study, in contrast, employed multi-centre, large-scale data from the Chinese population and demonstrated a clear inverse relationship between preoperative Tg levels and TNs malignancy, which was further validated by MR analysis, highlighting Tg's potential role not only as a postoperative surveillance marker but also as a preoperative diagnostic indicator. Tg is produced exclusively by well-differentiated thyroid follicular cells; its secretion remains high in benign nodules with intact tissue architecture, whereas in malignant nodules, reduced cellular differentiation impairs Tg synthesis and secretion 33. This decline in synthetic capacity may be reflected both locally (FNA-Tg) and systemically (serum Tg), with local detection offering potentially greater sensitivity. Thus, although some studies report elevated Tg levels in thyroid cancer (often associated with extensive follicular destruction or hypermetabolic states), our findings and prior FNA-Tg studies support that, in patients with well-preserved thyroid structure, the inverse correlation between Tg levels and malignancy risk aligns with underlying pathophysiological mechanisms. In line with the mechanisms, the inverse Tg-TSH correlation further reinforces Tg as a valid indicator of thyroid functional integrity.

Furthermore, multiple pieces of evidence support Tg as a key biomarker in differentiating malignant TNs. We found TG was significantly highly expressed in adjacent normal tissues when compared to tumour tissues, and high TG expression was strongly indicated with better survival outcomes, which also suggests that Tg levels are higher in preserved differentiation and functional integrity of thyroid follicular cells 21,23. Conversely, malignant nodules often exhibit reduced cellular differentiation, leading to decreased Tg synthesis and secretion 33. Further analysis based on transcriptomic and immune microenvironment profiling showed that high-TG expression samples were in unactivated immune environments often seen in benign tissues, whereas low-TG expression samples may reflect immune evasion environments in malignant tissues 34,35. These findings align with previous studies implicating Tg in immune regulation and tumor microenvironment remodelling.

Current international guidelines all recommend ultrasound as the preferred method for TNs risk assessment 36,37 Our study indicates that when serum Tg and age data were incorporated into the TI-RADS model, the model's predictive performance was further enhanced, suggesting that serum biomarkers can provide valuable supplementary information for imaging-based assessment. Unlike prior models relying heavily on invasive FNA (e.g., Bethesda) 38, whose performance is often operator-dependent, resource-intensive, and limited by accessibility—particularly in primary care or low-resource regions. The resulting two-factor model, based solely on age and Tg, is simple, low-cost, and easily applicable in clinical practice—particularly valuable in resource-limited settings as an adjunct to ultrasound, aiding clinical decision-making on whether to proceed with invasive procedures, thereby improving diagnostic efficiency and patient compliance.

To enhance practical utility, we also developed an interactive web-based nomogram, enabling intuitive, point-of-care risk estimation. Moreover, by integrating MR analysis, we offer new causal insights into thyroid biomarkers and TNs development, which has rarely been addressed in previous studies. However, several limitations should be acknowledged. First, our cohorts were recruited from southern China, a region with long-standing universal salt iodisation, which consequently limits the data available in iodine-deficient populations. Second, serum Tg levels may vary across different laboratory platforms, suggesting that the model may require standardisation or recalibration before application in other institutions. Therefore, we strongly encourage further validation studies in more diverse populations to improve the generalizability and clinical utility of the model.

In conclusion, our large-scale retrospective study successfully constructed and validated a two-factor model for diagnosing malignant TNs only through routine thyroid function tests. With robust performance and wide applicability, this model holds promise as a non-invasive complementary tool to existing ultrasound-based risk stratification systems, helping to reduce unnecessary procedures and alleviate healthcare burden.

## Conclusion

In this study, we developed and validated a simple two-factor model based on age and serum Tg for preoperative risk stratification of TNs. The model demonstrated robust and consistent performance across multiple validation cohorts and clinically relevant subgroups, and demonstrated incremental value to TI-RADS. Multilevel validation using MR and transcriptomic analyses supported the diagnostic relevance and biological plausibility of serum Tg. Collectively, this model provides a complementary tool to existing ultrasound-based risk stratification systems and may assist risk stratification in settings where invasive procedures or resource-intensive assessments are less accessible.

## Supplementary Material

Supplementary figures and tables.

## Figures and Tables

**Table 1:** Demographic characteristics and thyroid biomarkers of the overall cohort and subgroup cohorts based on malignancy of thyroid nodules.

Variable	Overall	benign	malignant	P value*^2^*
	N = 5,537*^1^*	N = 1,870*^1^*	N = 3,667*^1^*	
**Sex**				0.261
Female	4,058 (73%)	1,388 (74%)	2,670 (73%)	
Male	1,479 (27%)	482 (26%)	997 (27%)	
**Age, y**	43 (34, 52)	47 (38, 56)	41 (33, 49)	<0.001
**FT3, pmol/L**	4.80 (4.40, 5.21)	4.82 (4.40, 5.21)	4.80 (4.39, 5.20)	0.496
**FT4, pmol/L**	11.20 (10.21, 12.39)	11.20 (10.22, 12.44)	11.20 (10.20, 12.35)	0.795
**TT3, nmol/L**	1.52 (1.34, 1.71)	1.53 (1.34, 1.73)	1.51 (1.34, 1.69)	0.002
**TT4, nmol/L**	104 (92, 117)	105 (92, 117)	104 (93, 116)	0.852
**TSH, mIU/L**	1.49 (0.95, 2.29)	1.24 (0.75, 1.90)	1.64 (1.07, 2.48)	<0.001
**Tg, ng/mL**	19 (7, 54)	43 (16, 146)	14 (5, 32)	<0.001
**TGAB, IU/mL**				<0.001
≤40	4,493 (90%)	1,647 (95%)	2,846 (88%)	
>40	474 (9.5%)	89 (5.1%)	385 (12%)	
**TPOAb, IU/mL**				<0.001
≤35	4,158 (84%)	1,549 (89%)	2,609 (81%)	
>35	809 (16%)	187 (11%)	622 (19%)	
**Postop-hospital Stay, day**	2.00 (2.00, 3.00)	2.00 (1.00, 2.00)	2.00 (2.00, 3.00)	<0.001
**Total Fee, yuan (¥)**	17,073 (14,023, 20,988)	15,387 (12,897, 18,092)	18,488 (14,977, 22,224)	<0.001
**Operation Fee, yuan (¥)**	4,212 (2,890, 5,954)	3,335 (2,550, 4,224)	4,550 (3,335, 6,420)	<0.001

1. Median (IQR) or Frequency (%), 2. Wilcoxon rank sum test; Pearson's Chi-squared test

**Table 2:** Stepwise logistic regression modeling and predictive performance across training, internal validation, and external validation cohorts.

	Models, Odds Ratio (95% CI)^ 1^			
	1.ALL Variables	2.Gender, Age, TT3, TT4, TSH, Tg, TPOAb	3.Age, TSH, Tg	4.Age, Tg
**Predictors**				
Gender				
Female	1 [Reference]			
Male	1.35 (1.12-1.63)	1.33 (1.11-1.6)		
Age, years	0.96 (0.95-0.96)	0.96 (0.95-0.97)	0.96 (0.95-0.97)	0.96 (0.95-0.97)
FT3, pmol/L	0.98 (0.85-1.13)			
FT4, pmol/L	0.95 (0.9-1)			
TT3, nmol/L	0.64 (0.46-0.9)	0.66 (0.49-0.89)		
TT4, nmol/L	1.01 (1-1.02)	1.01 (1-1.01)		
TSH, uIU/mL	1.08 (1.02-1.14)	1.09 (1.03-1.15)	1.07 (1.02-1.13)	
lnTg, ng/mL	0.64 (0.61-0.67)	0.64 (0.61-0.68)	0.64 (0.61-0.68)	0.64 (0.61-0.67)
TGAB, IU/mL				
≤40	1 [Reference]			
>40	0.73 (0.51-1.06)			
TPOAb, IU/mL				
≤35	1 [Reference]			
>35	1.12 (0.87-1.45)			
**Model Performance Measures ^2^**				
AIC	3,604.95	3,604.24	3,617.91	3,625.31
**Training Cohort**				
AUC	0.763 (0.745-0.78)	0.762 (0.745-0.779)	0.758 (0.74-0.775)	0.755 (0.737-0.772)
IDI, %	-	-0.001	-0.005	-0.008
*P* value^ 3^	-	0.782	0.030	0.004
**Internal Validation Cohort**				
AUC	0.743 (0.715-0.77)	0.743 (0.716-0.77)	0.742 (0.715-0.769)	0.735 (0.708-0.762)
IDI, %	-	0	-0.001	-0.008
*P* value^ 3^	-	0.759	0.881	0.048
**External Validation Cohort**				
AUC	0.786 (0.732-0.839)	0.784 (0.731-0.838)	0.786 (0.733-0.839)	0.784 (0.731-0.837)
IDI, %	-	-0.002	0	-0.002
*P* value^ 3^	-	0.729	0.956	0.776

1. Variables were selected through stepwise logistic regression, and corresponding odds ratios (OR) with 95% confidence intervals (CI) are reported. 2. Model performance was assessed by Akaike Information Criterion (AIC), Area Under the Curve (AUC), and Integrated Discrimination Improvement (IDI). 3. Differences in AUC were tested by DeLong's test.

## Data Availability

The complete datasets (each containing only key variables: “Benign_vs_Malignant”, “Age”, and “lnTg”), R code for model development and validation, and the trained logistic regression model are publicly available in the GitHub repository: https://github.com/Shirline2024/tg-age-thyroid-risk-model. Due to ethical and privacy restrictions, the full individual-level data for the cohorts cannot be made publicly available. Instead, we provide detailed descriptive statistics for these datasets to facilitate methodological transparency. Researchers interested in accessing the full datasets may contact the corresponding author and submit a reasonable request in compliance with institutional review board guidelines.

## Abbreviations

TNs: Thyroid nodules
FNA: Fine-needle aspiration
TSH: Thyroid-stimulating hormone
T3: Triiodothyronine
T4: Thyroxine
T3: Free triiodothyronine
T4: Free thyroxine
Tg: Thyroglobulin
TGAB: Anti-thyroglobulin antibody
TPOAb: Thyroid peroxidase antibody
LR: Logistic regression
RF: Random Forest
GBDT: Gradient Boosting Decision Tree
XGBoost: eXtreme Gradient Boosting
SVM: Support Vector Machine
SHAP: SHapley Additive exPlanations
AUC: The area under the receiver operating characteristic curve
AIC: Akaike Information Criterion
IDI: Integrated discrimination improvement
NHANES: U.S. National Health and Nutrition Examination Survey
MR: Mendelian Randomization
IVW: Inverse-variance weighted
GWAS: Genome-Wide Association Study
TCGA: The Cancer Genome Atlas
DGEs: Differential gene expression
GO: Gene Ontology
KEGG: Kyoto Encyclopedia of Genes and Genomes
GSEA: Gene Set Enrichment Analysis
IQR: Interquartile range
ln: Natural logarithm
ECM: Extracellular matrix
