# Supplementary material for: Serum Thyroid Biomarkers for Diagnosing Malignant Thyroid Nodules: A Machine Learning Approach with External and Causal Validation
Source: J Cancer. 2026 May 18;17(5):1082–93. doi: 10.7150/jca.131422 (PMC13190228; doi:10.7150/jca.131422)
Supplement: Supplementary file 1 — Supplementary figures and tables. [file jcav17p1082s1.pdf]

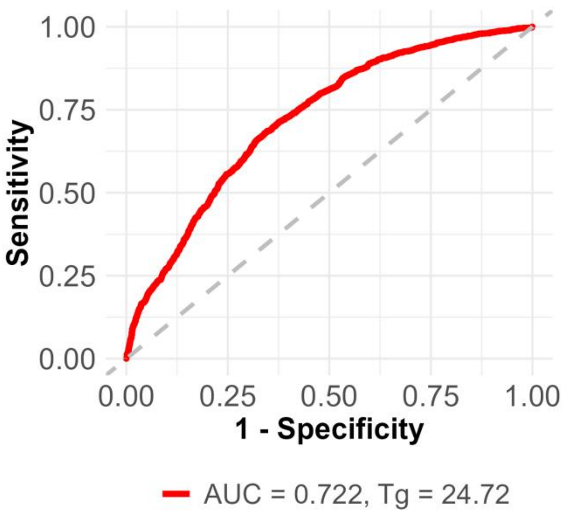

Supplemental Figure 1. ROC curve of serum thyroglobulin (Tg) level for differentiating malignant and benign nodules.

The optimal cutoff value of Tg is 24.72 ng/mL, and the area under the curve (AUC) is 0.722.

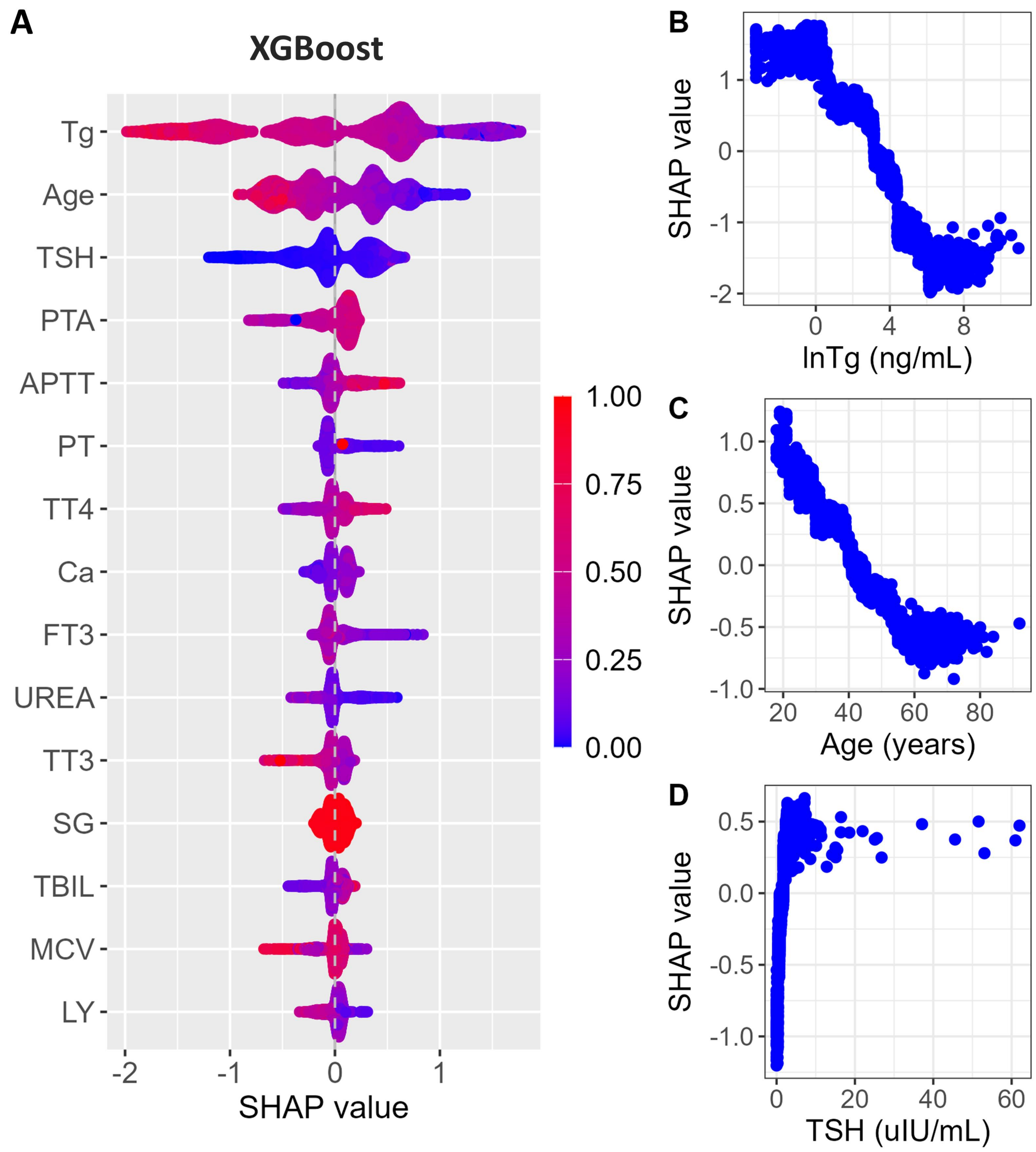

Supplemental Figure 2. SHapley Additive exPlanations (SHAP) summary plot illustrating the contribution of top features to the prediction of thyroid nodule malignancy using the Extreme Gradient Boosting (XGBoost) model.

A. Summary plot ranking candidate variables by their importance (y-axis) and showing their relationship with malignancy.

B-C. SHAP dependence plots for the top three features. Higher SHAP values indicate a greater contribution to the predicted malignancy risk.

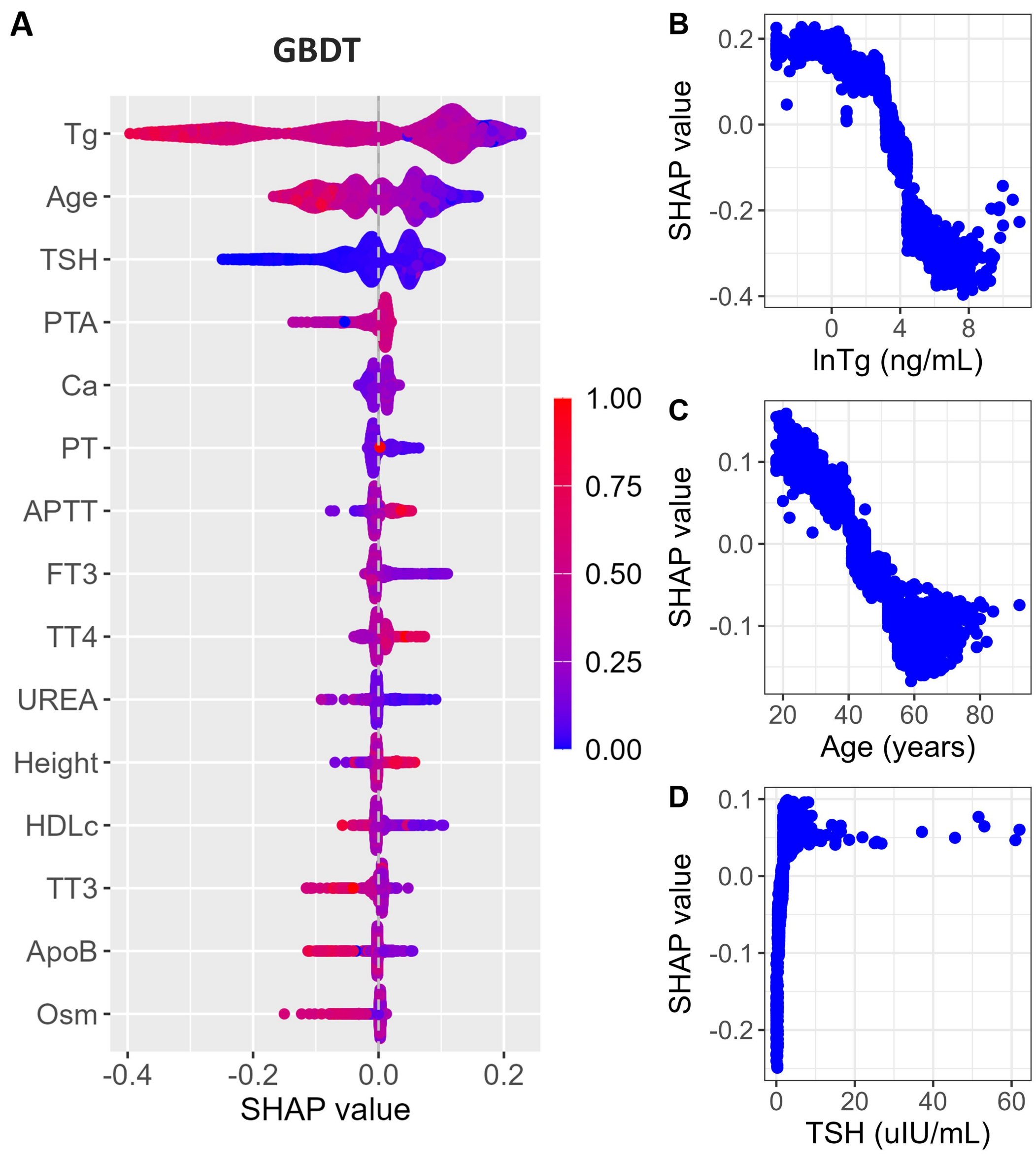

Supplemental Figure 3. SHapley Additive exPlanations (SHAP) summary plot illustrating the contribution of top features to the prediction of thyroid nodule malignancy using the Gradient Boosting Decision Tree (GBDT) model.

A. Summary plot ranking candidate variables by their importance (y-axis) and showing their relationship with malignancy.

B-C. SHAP dependence plots for the top three features. Higher SHAP values indicate a greater contribution to the predicted malignancy risk.

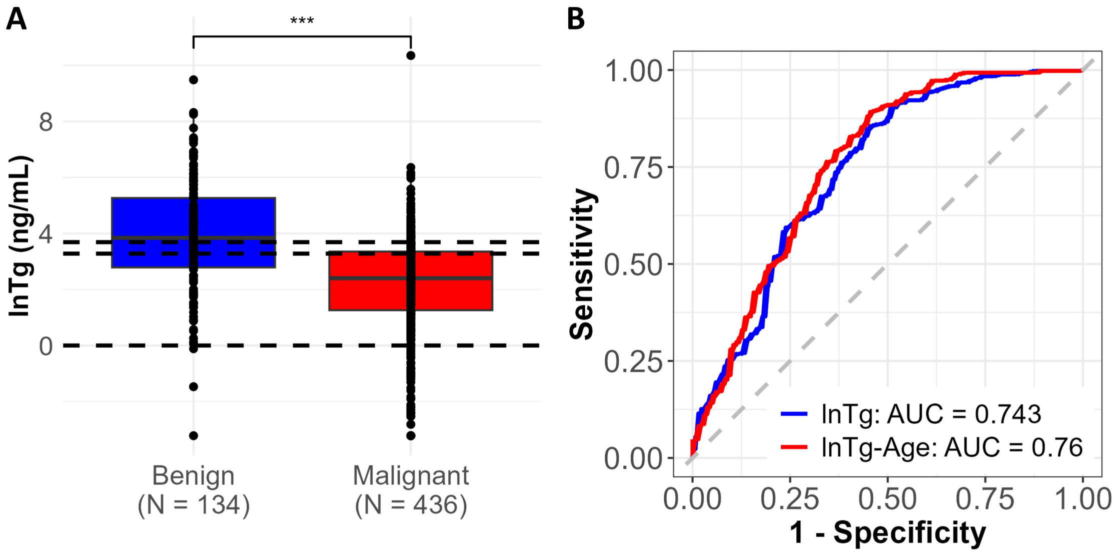

Supplemental Figure 4. Distribution and diagnostic performance of key factors in the temporal validation cohort.

A. The distribution of thyroglobulin (Tg) across benign and malignant nodules within the additional external validation dataset. The black dashed lines show Tg = 1, 26.58, and 40 ng/mL. The statistical significance computed by the Wilcoxon test is annotated by the number of stars (***: P value <0.001).

B. The receiver operating characteristic (ROC) curves of Tg alone and Tg combined with age in the additional external validation cohort. The blue line shows Tg-only model, while the red line shows Tg-age model.

Supplemental Table 1. Comparison of clinical variables between benign and malignant thyroid nodules and results of univariable logistic regression analysis.

**Variable**	**N**	**Overall[1]**	**Benign[1]**	**Malignant[1]**	**p-value[1]**	**FDR[2]**	**OR (95%CI)[3]**	**AUC[4]**	**Logistic p-value[5]**
Gender, Female	4,668	3,406 (73%)	1,204 (74%)	2,202 (72%)	0.116	0.201	1.12 (0.97-1.28)	0.511 (0.497-0.524)	0.116
Age, y	4,668	43 (34, 51)	47 (38, 56)	40 (32, 49)	<0.001	<0.001	0.96 (0.95-0.96)	0.64 (0.624-0.657)	0.000
Height, cm	3,992	161 (158, 168)	160 (157, 166)	161 (158, 168)	<0.001	<0.001	1.02 (1.01-1.03)	0.542 (0.523-0.561)	0.000
Weight, kg	4,519	60 (53, 67)	59 (53, 66)	60 (53, 68)	0.073	0.134	1.01 (1-1.01)	0.516 (0.499-0.534)	0.008
BMI, kg/m²	3,989	22.7 (20.6, 25.0)	22.8 (20.8, 24.9)	22.7 (20.6, 25.0)	0.488	0.593	1 (0.98-1.02)	0.507 (0.488-0.525)	0.947
HR, bpm	4,394	79 (72, 84)	79 (72, 84)	80 (72, 84)	0.723	0.813	1 (0.99-1)	0.497 (0.479-0.515)	0.609
SBP, mmHg	4,431	117 (109, 127)	119 (110, 130)	116 (108, 126)	<0.001	<0.001	0.99 (0.98-0.99)	0.544 (0.526-0.562)	0.000
DBP, mmHg	4,431	75 (69, 80)	75 (69, 81)	75 (69, 80)	0.12	0.201	1 (0.99-1)	0.514 (0.496-0.532)	0.193
MAP, mmHg	4,431	89 (83, 96)	90 (83, 97)	89 (83, 95)	<0.001	0.002	0.99 (0.98-1)	0.531 (0.513-0.549)	0.001
Free Triiodothyronine (FT3), pmol/L	4,668	4.83 (4.45, 5.25)	4.83 (4.49, 5.27)	4.83 (4.43, 5.25)	0.354	0.456	0.95 (0.86-1.04)	0.508 (0.491-0.526)	0.235
Free Thyroxine (FT4), pmol/L	4,668	11.20 (10.19, 12.28)	11.20 (10.17, 12.32)	11.20 (10.20, 12.23)	0.773	0.839	1 (0.96-1.03)	0.503 (0.485-0.52)	0.829
Total Triiodothyronine (TT3), nmol/L	4,668	1.52 (1.35, 1.72)	1.54 (1.36, 1.74)	1.52 (1.34, 1.71)	0.002	0.005	0.7 (0.57-0.86)	0.528 (0.51-0.545)	0.001
Total Thyroxine (TT4), nmol/L	4,668	105 (93, 117)	105 (93, 118)	105 (93, 117)	0.748	0.821	1 (1-1)	0.503 (0.485-0.52)	0.597
Thyroid-Stimulating	4,668	1.51 (0.97, 2.31)	1.25 (0.75, 1.93)	1.65 (1.08, 2.50)	<0.001	<0.001	1.21 (1.15-1.27)	0.62 (0.603-0.637)	0.000

Hormone (TSH), mIU/L									
Thyroglobulin (lnTg), ng/mL	4,668	2.99 (2.00, 4.09)	3.78 (2.80, 5.02)	2.66 (1.66, 3.52)	<0.001	<0.001	0.64 (0.61-0.66)	0.719 (0.703-0.734)	0.000
Thyroglobulin Antibody (TGAB), pos	4,668	428 (9.2%)	74 (4.6%)	354 (12%)	<0.001	<0.001	2.74 (2.12-3.55)	0.535 (0.528-0.543)	0.000
Thyroid Peroxidase Antibody (TPOAb), pos	4,668	760 (16%)	170 (11%)	590 (19%)	<0.001	<0.001	2.05 (1.7-2.45)	0.544 (0.534-0.554)	0.000
Hypertension, Yes	4,668	444 (9.5%)	201 (12%)	243 (8.0%)	<0.001	<0.001	0.61 (0.5-0.74)	0.522 (0.513-0.532)	0.000
Diabetes, Yes	4,668	156 (3.3%)	62 (3.8%)	94 (3.1%)	0.177	0.265	0.8 (0.58-1.11)	0.504 (0.498-0.509)	0.178
Smoking/Alcohol History, Yes	4,668	298 (6.4%)	112 (6.9%)	186 (6.1%)	0.277	0.395	0.87 (0.69-1.11)	0.504 (0.497-0.512)	0.277
Heart Diseases, Yes	4,668	93 (2.0%)	46 (2.8%)	47 (1.5%)	0.002	0.006	0.54 (0.35-0.81)	0.506 (0.502-0.511)	0.003
Autoimmune Disease, Yes	4,668	95 (2.0%)	28 (1.7%)	67 (2.2%)	0.281	0.395	1.28 (0.82-1.99)	0.502 (0.498-0.506)	0.282
Hepatitis B, Yes	4,668	421 (9.0%)	152 (9.4%)	269 (8.8%)	0.521	0.601	0.93 (0.76-1.15)	0.503 (0.494-0.512)	0.521
Calcitonin, ng/L	3,493	2.00 (2.00, 2.00)	2.00 (2.00, 2.00)	2.00 (2.00, 2.00)	<0.001	0.002	1.01 (0.99-1.02)	0.469 (0.452-0.486)	0.313
Carcinoembryonic Antigen (CEA), μg/mL	2,771	1.33 (0.90, 1.94)	1.37 (0.91, 2.01)	1.31 (0.90, 1.91)	0.053	0.106	1 (0.99-1.01)	0.522 (0.5-0.545)	0.542
Thyrotropin Receptor Antibody (TRAb), IU/L	178	0.30 (0.30, 0.39)	0.30 (0.30, 0.42)	0.30 (0.30, 0.36)	0.353	0.456	1.18 (0.75-1.86)	0.462 (0.381-0.543)	0.465
Alpha-Fetoprotein	52	2.34 (1.96, 3.33)	2.51 (1.93, 3.19)	2.34 (1.96, 3.46)	0.843	0.892	1.11 (0.6-2.08)	0.482 (0.305-0.658)	0.735

(AFP), ng/mL									
Cancer Antigen 15-3 (CA15-3), U/mL	50	8.1 (5.6, 10.2)	9.6 (5.6, 10.2)	7.2 (5.6, 9.9)	0.512	0.601	0.92 (0.81-1.04)	0.558 (0.373-0.743)	0.189
Cancer Antigen 19-9 (CA19-9), U/mL	52	8 (4, 14)	8 (4, 14)	8 (3, 15)	0.977	0.977	0.99 (0.94-1.04)	0.503 (0.326-0.681)	0.708
Alanine Aminotransferase (ALT), U/L	4,624	15 (11, 21)	15 (11, 21)	15 (11, 22)	0.378	0.472	1 (1-1)	0.492 (0.475-0.509)	0.620
Aspartate Aminotransferase (AST), U/L	4,624	19.0 (16.0, 22.0)	19.0 (16.0, 22.0)	18.0 (16.0, 22.0)	0.003	0.008	1 (0.99-1)	0.526 (0.509-0.544)	0.145
Alkaline Phosphatase (ALP), U/L	4,624	66 (57, 77)	68 (58, 79)	65 (56, 75)	<0.001	<0.001	0.99 (0.99-0.99)	0.553 (0.535-0.57)	0.000
Total Protein (TP), g/L	4,626	68.9 (65.3, 72.6)	68.5 (64.9, 72.1)	69.1 (65.5, 72.9)	<0.001	0.003	1.02 (1.01-1.03)	0.529 (0.512-0.547)	0.001
Albumin (ALB), g/L	4,626	41.6 (39.5, 44.0)	41.3 (39.1, 43.7)	41.9 (39.7, 44.2)	<0.001	<0.001	1.05 (1.03-1.07)	0.544 (0.526-0.561)	0.000
Globulin (GLB), g/L	4,625	27.2 (24.5, 29.7)	27.2 (24.5, 29.5)	27.1 (24.5, 29.7)	0.899	0.919	1 (0.99-1.02)	0.499 (0.481-0.516)	0.751
Total Bilirubin (TBIL), μmol/L	4,625	11.1 (8.9, 14.3)	10.9 (8.8, 14.0)	11.2 (8.9, 14.4)	0.156	0.246	1.01 (1-1.03)	0.513 (0.495-0.53)	0.027
Calcium (Ca), mmol/L	4,618	2.27 (2.20, 2.33)	2.26 (2.20, 2.32)	2.28 (2.20, 2.33)	<0.001	<0.001	3.06 (1.71-5.48)	0.537 (0.519-0.554)	0.000
Phosphorus (PHOS), mmol/L	4,602	1.19 (1.08, 1.30)	1.19 (1.08, 1.30)	1.19 (1.08, 1.29)	0.895	0.919	0.97 (0.67-1.4)	0.501 (0.484-0.519)	0.855
Sodium (Na), mmol/L	4,626	140.00 (139.00, 141.00)	140.00 (139.00, 141.00)	140.00 (139.00, 141.00)	<0.001	<0.001	0.92 (0.89-0.95)	0.537 (0.52-0.555)	0.000

Potassium (K), mmol/L	4,626	3.99 (3.83, 4.17)	3.98 (3.81, 4.17)	4.00 (3.83, 4.17)	0.156	0.246	1.13 (0.91-1.4)	0.513 (0.495-0.53)	0.278
Chloride (Cl), mmol/L	4,626	106.00 (104.00, 107.00)	106.00 (104.00, 107.00)	106.00 (104.00, 107.00)	<0.001	0.003	0.95 (0.93-0.98)	0.529 (0.512-0.546)	0.002
Carbon Dioxide (CO2), mmol/L	4,626	25.00 (24.00, 27.00)	26.00 (24.00, 27.00)	25.00 (24.00, 27.00)	<0.001	<0.001	0.95 (0.92-0.97)	0.534 (0.517-0.551)	0.000
Glucose (GLU), mmol/L	4,626	4.70 (4.40, 5.10)	4.70 (4.40, 5.10)	4.70 (4.30, 5.00)	<0.001	<0.001	0.83 (0.77-0.9)	0.54 (0.522-0.557)	0.000
Urea (UREA), mmol/L	4,626	4.40 (3.70, 5.20)	4.50 (3.90, 5.30)	4.40 (3.70, 5.10)	<0.001	<0.001	0.88 (0.84-0.92)	0.552 (0.535-0.569)	0.000
Creatinine (CREA), µmol/L	4,626	62 (54, 74)	61 (54, 73)	62 (54, 74)	0.363	0.46	1 (1-1)	0.508 (0.491-0.526)	0.928
Uric Acid (UA), µmol/L	4,611	329 (280, 396)	327 (281, 393)	331 (279, 399)	0.316	0.427	1 (1-1)	0.509 (0.492-0.526)	0.110
Anion Gap (AG)	4,626	13.00 (12.00, 14.00)	13.00 (11.00, 14.00)	13.00 (12.00, 14.00)	<0.001	0.002	1.05 (1.02-1.09)	0.531 (0.514-0.548)	0.000
Osmolality (Osm), mOsm/kg	4,626	289.0 (287.0, 292.0)	290.0 (287.0, 293.0)	289.0 (286.0, 292.0)	<0.001	<0.001	0.95 (0.93-0.96)	0.559 (0.541-0.576)	0.000
Cholesterol (CHOL), mmol/L	3,795	4.70 (4.20, 5.40)	4.80 (4.20, 5.50)	4.70 (4.10, 5.40)	<0.001	<0.001	0.86 (0.8-0.92)	0.543 (0.524-0.562)	0.000
Triglycerides (TG), mmol/L	3,758	1.06 (0.76, 1.54)	1.09 (0.79, 1.59)	1.05 (0.76, 1.52)	0.005	0.013	0.93 (0.87-1)	0.528 (0.508-0.547)	0.041
Low-Density Lipoprotein Cholesterol (LDLc), mmol/L	3,795	2.93 (2.50, 3.46)	2.97 (2.56, 3.52)	2.91 (2.47, 3.43)	<0.001	0.003	0.85 (0.78-0.94)	0.533 (0.513-0.552)	0.001

High-Density Lipoprotein Cholesterol (HDLc), mmol/L	3,795	1.22 (1.05, 1.43)	1.26 (1.07, 1.47)	1.21 (1.04, 1.41)	<0.001	<0.001	0.62 (0.49-0.78)	0.543 (0.524-0.562)	0.000
Apolipoprotein A1 (ApoA1), g/L	3,795	1.28 (1.15, 1.42)	1.30 (1.18, 1.46)	1.26 (1.13, 1.40)	<0.001	<0.001	0.41 (0.3-0.56)	0.558 (0.539-0.577)	0.000
Apolipoprotein B (ApoB), g/L	3,795	0.83 (0.70, 0.98)	0.85 (0.72, 1.01)	0.82 (0.68, 0.97)	<0.001	<0.001	0.43 (0.31-0.59)	0.551 (0.532-0.57)	0.000
Apolipoprotein E (ApoE), mg/L	3,795	39 (33, 47)	40 (33, 48)	39 (33, 46)	0.008	0.017	0.99 (0.99-1)	0.526 (0.507-0.546)	0.007
Lipoprotein(a) (LPa), mg/L	3,795	98 (52, 223)	105 (56, 239)	95 (50, 216)	0.005	0.011	1 (1-1)	0.528 (0.509-0.547)	0.022
White Blood Cell Count (WBC), $\times 10^9$/L	4,629	5.96 (5.08, 7.01)	5.95 (5.09, 7.02)	5.97 (5.07, 7.01)	0.834	0.892	0.99 (0.96-1.03)	0.498 (0.481-0.516)	0.751
Neutrophils (NEUT), $\times 10^9$/L	4,629	3.30 (2.66, 4.06)	3.29 (2.66, 4.08)	3.31 (2.66, 4.05)	0.51	0.601	1.01 (0.96-1.06)	0.506 (0.488-0.523)	0.695
Lymphocytes (LY), $\times 10^9$/L	4,629	2.00 (1.65, 2.38)	2.00 (1.65, 2.40)	2.00 (1.65, 2.36)	0.429	0.529	0.96 (0.87-1.07)	0.507 (0.49-0.525)	0.495
Monocytes (MO), $\times 10^9$/L	4,629	0.41 (0.33, 0.50)	0.41 (0.33, 0.50)	0.41 (0.33, 0.51)	0.732	0.814	0.85 (0.57-1.28)	0.503 (0.486-0.52)	0.438
Eosinophils (EO), $\times 10^9$/L	4,629	0.12 (0.09, 0.20)	0.12 (0.09, 0.20)	0.12 (0.09, 0.20)	0.262	0.381	0.66 (0.41-1.05)	0.51 (0.493-0.527)	0.076
Basophils (BASO), $\times 10^9$/L	4,629	0.03 (0.01, 0.04)	0.03 (0.01, 0.04)	0.03 (0.01, 0.04)	0.073	0.134	0.14 (0.02-1.06)	0.516 (0.499-0.533)	0.057
Red Blood Cell	4,629	4.57 (4.25, 4.95)	4.55 (4.23, 4.92)	4.58 (4.26, 4.96)	0.175	0.265	1.08 (0.97-1.2)	0.512 (0.495-0.53)	0.178

Count (RBC), ×10¹²/L									
Hemoglobin (Hb), g/L	4,634	133 (124, 144)	133 (124, 143)	133 (124, 144)	0.16	0.249	1 (1-1.01)	0.513 (0.495-0.53)	0.142
Hematocrit (Ht), %	4,629	0.40 (0.37, 0.43)	0.40 (0.37, 0.43)	0.40 (0.37, 0.43)	0.318	0.427	2.14 (0.52-8.92)	0.509 (0.492-0.526)	0.294
Mean Corpuscular Hemoglobin Concentration (MCHC), g/L	4,629	334 (327, 341)	334 (327, 340)	334 (327, 341)	0.056	0.109	1 (1-1.01)	0.517 (0.5-0.534)	0.106
Mean Corpuscular Hemoglobin (MCH), pg	4,629	29.7 (28.4, 30.7)	29.6 (28.3, 30.8)	29.7 (28.4, 30.7)	0.717	0.813	1 (0.98-1.02)	0.503 (0.486-0.521)	0.952
Mean Corpuscular Volume (MCV), fL	4,629	88 (85, 91)	88 (85, 91)	88 (85, 91)	0.342	0.452	1 (0.99-1.01)	0.508 (0.491-0.526)	0.519
Red Blood Cell Distribution Width CV (RDW), %	4,628	0.130 (0.120, 0.140)	0.130 (0.120, 0.140)	0.130 (0.120, 0.130)	<0.001	<0.001	0.03 (0-1.62)	0.534 (0.518-0.55)	0.086
Platelet Count (PLT), ×10⁹/L	4,634	238 (203, 279)	236 (202, 277)	239 (204, 280)	0.115	0.201	1 (1-1)	0.514 (0.497-0.531)	0.130
Plateletcrit (PCT), %	4,530	0.24 (0.20, 0.28)	0.23 (0.19, 0.28)	0.24 (0.20, 0.28)	<0.001	0.002	5.27 (1.93-14.42)	0.531 (0.514-0.549)	0.001
Mean Platelet Volume (MPV), fL	4,530	10.10 (9.23, 10.80)	10.00 (9.11, 10.70)	10.10 (9.30, 10.80)	<0.001	<0.001	1.11 (1.05-1.17)	0.535 (0.517-0.552)	0.000
Platelet Distribution Width (PDW), %	4,530	12.70 (11.20, 16.10)	12.60 (11.20, 16.08)	12.70 (11.30, 16.10)	0.295	0.408	1.01 (0.99-1.04)	0.509 (0.492-0.527)	0.318
Prothrombin Time (PT), s	4,628	11.20 (10.70, 11.70)	11.20 (10.80, 11.70)	11.20 (10.70, 11.70)	0.517	0.601	0.99 (0.94-1.06)	0.506 (0.489-0.523)	0.855

Prothrombin Time Activity (PTA), %	4,628	102 (97, 108)	102 (97, 108)	102 (97, 108)	0.077	0.139	1 (1-1.01)	0.516 (0.498-0.533)	0.180
International Normalized Ratio (INR)	4,628	0.96 (0.91, 1.00)	0.95 (0.91, 1.00)	0.96 (0.91, 1.00)	0.868	0.909	1.02 (0.49-2.12)	0.501 (0.484-0.519)	0.964
Activated Partial Thromboplastin Time (APTT), s	4,627	28.9 (26.5, 31.7)	28.6 (26.3, 31.1)	29.1 (26.7, 31.9)	<0.001	<0.001	1.04 (1.02-1.05)	0.541 (0.524-0.559)	0.000
Thrombin Time (TT), s	4,628	17.60 (17.00, 18.40)	17.70 (17.00, 18.40)	17.60 (17.00, 18.30)	0.009	0.02	0.97 (0.92-1.01)	0.523 (0.506-0.541)	0.122
Fibrinogen (Fbg), g/L	4,628	2.49 (2.17, 2.91)	2.54 (2.22, 2.96)	2.47 (2.14, 2.87)	<0.001	<0.001	0.78 (0.71-0.86)	0.543 (0.525-0.56)	0.000
Urine Specific Gravity (SG)	4,535	1.020 (1.015, 1.024)	1.019 (1.014, 1.024)	1.020 (1.015, 1.025)	<0.001	<0.001	27.13 (0.23-3243.5)	0.543 (0.525-0.56)	0.176
Urinary Granulocyte Esterase (UGE), pos	4,540	1,290 (28%)	472 (30%)	818 (28%)	0.12	0.201	0.9 (0.79-1.03)	0.511 (0.497-0.525)	0.120
Urinary Nitrite (UNIT), pos	4,540	75 (1.7%)	35 (2.2%)	40 (1.4%)	0.03	0.065	0.61 (0.38-0.96)	0.504 (0.5-0.508)	0.032
Urinary Glucose (UG), pos	4,540	39 (0.9%)	18 (1.1%)	21 (0.7%)	0.137	0.224	0.62 (0.33-1.17)	0.502 (0.499-0.505)	0.140
Urinary Protein (UP), pos	4,540	420 (9.3%)	146 (9.2%)	274 (9.3%)	0.97	0.977	1 (0.81-1.24)	0.5 (0.491-0.509)	0.970
Urinary Ketone Bodies (UKB), pos	4,540	246 (5.4%)	77 (4.9%)	169 (5.7%)	0.23	0.34	1.18 (0.9-1.56)	0.504 (0.497-0.511)	0.231
Urobilinogen (URO), pos	4,481	76 (1.7%)	18 (1.2%)	58 (2.0%)	0.043	0.089	1.72 (1.01-2.94)	0.504 (0.5-0.508)	0.045

Urobilinogen (UBG), pos	4,540	31 (0.7%)	6 (0.4%)	25 (0.8%)	0.069	0.133	2.24 (0.92-5.47)	0.502 (0.5-0.505)	0.077
Urine Occult Blood (UOB), pos	4,540	1,274 (28%)	473 (30%)	801 (27%)	0.044	0.09	0.87 (0.76-1)	0.514 (0.5-0.528)	0.044
Urinary Epithelial Cells (UEC), pos	4,434	1,508 (34%)	478 (31%)	1,030 (35%)	0.006	0.013	1.2 (1.06-1.37)	0.521 (0.506-0.535)	0.006
Urine Bacteria (BACT), pos	4,530	1,258 (28%)	394 (25%)	864 (29%)	0.002	0.006	1.24 (1.08-1.42)	0.521 (0.508-0.535)	0.002

1. Continuous variables are presented as median (interquartile range) and compared by the Wilcoxon rank-sum test; categorical variables are presented as counts (percentages) and compared by the Chi-squared test.

2. P values for group comparisons were adjusted by the false discovery rate (FDR) correction.

3. Odds ratios (OR) and 95% confidence intervals (CI) are derived from univariable logistic regression.

4. The predictive performance of the logistic regression model was further evaluated using the area under the receiver operating characteristic curve (AUC).

5. A $P < 0.05$ was considered statistically significant for univariable logistic regression.

Supplemental Table 2. Baseline characteristics of the subgroups cohort for both the training set and test set.

	Training Set				Test Set			
	**Benign**	**Malignant**			**Benign**	**Malignant**		
**Variable**	N = 1,133[1]	N = 2,134[1]	**p-value[2]**	**FDR[3]**	N = 486[1]	N = 915[1]	**p-value[2]**	**FDR[3]**
Gender			0.136	0.255			0.555	0.654
Female	840 (74%)	1,530 (72%)			364 (75%)	672 (73%)		
Male	293 (26%)	604 (28%)			122 (25%)	243 (27%)		
Age, y	47 (38, 57)	40 (32, 49)	<0.001	<0.001	46 (38, 55)	40 (32, 49)	<0.001	<0.001
Height, cm	161 (158, 165)	161 (158, 167)	<0.001	<0.001	161 (158, 165)	161 (158, 166)	0.112	0.235
Weight, kg	60 (53, 67)	60 (53, 68)	0.241	0.369	59 (52, 65)	60 (53, 67)	0.149	0.262
BMI, kg/m²	23.1 (20.8, 25.0)	22.8 (20.6, 25.1)	0.165	0.28	22.4 (20.4, 24.3)	22.7 (20.5, 24.9)	0.178	0.292
HR, bpm	79 (72, 84)	79 (72, 82)	0.928	0.963	80 (75, 84)	79 (74, 84)	0.511	0.613
SBP, mmHg	118 (110, 129)	117 (110, 125)	<0.001	<0.001	117 (110, 128)	116 (108, 124)	0.006	0.026
DBP, mmHg	75 (70, 81)	75 (70, 80)	0.316	0.442	75 (70, 80)	75 (69, 80)	0.195	0.309
MAP, mmHg	89 (84, 96)	89 (83, 95)	0.007	0.021	89 (83, 96)	89 (83, 93)	0.038	0.105
FT3, pmol/L	4.83 (4.50, 5.26)	4.83 (4.43, 5.26)	0.529	0.635	4.83 (4.46, 5.27)	4.83 (4.42, 5.20)	0.478	0.582
FT4, pmol/L	11.20 (10.17, 12.39)	11.20 (10.20, 12.20)	0.529	0.635	11.20 (10.15, 12.26)	11.20 (10.19, 12.29)	0.666	0.737
TT3, nmol/L	1.54 (1.36, 1.74)	1.52 (1.34, 1.71)	0.005	0.017	1.53 (1.35, 1.75)	1.52 (1.34, 1.70)	0.155	0.266
TT4, nmol/L	105 (93, 119)	105 (94, 117)	0.667	0.758	104 (93, 116)	104 (93, 115)	0.949	0.949
TSH, mIU/L	1.23 (0.74, 1.95)	1.66 (1.09, 2.48)	<0.001	<0.001	1.27 (0.79, 1.89)	1.65 (1.06, 2.55)	<0.001	<0.001
lnTg, ng/mL	3.80 (2.82, 4.99)	2.66 (1.66, 3.49)	<0.001	<0.001	3.74 (2.78, 5.05)	2.65 (1.68, 3.60)	<0.001	<0.001
TGAB, IU/mL			<0.001	<0.001			<0.001	<0.001
≤40	1,080 (95%)	1,883 (88%)			465 (96%)	812 (89%)		
>40	53 (4.7%)	251 (12%)			21 (4.3%)	103 (11%)		
TPOAb, IU/mL			<0.001	<0.001			<0.001	<0.001

≤35	1,010 (89%)	1,717 (80%)			439 (90%)	742 (81%)		
>35	123 (11%)	417 (20%)			47 (9.7%)	173 (19%)		
Hypertension, Yes	148 (13%)	174 (8.2%)	<0.001	<0.001	53 (11%)	69 (7.5%)	0.034	0.1
Diabetes, Yes	46 (4.1%)	68 (3.2%)	0.195	0.322	16 (3.3%)	26 (2.8%)	0.638	0.724
Tobacco and alcohol History, Yes	80 (7.1%)	137 (6.4%)	0.484	0.598	32 (6.6%)	49 (5.4%)	0.348	0.464
Heart Diseases, Yes	30 (2.6%)	33 (1.5%)	0.029	0.07	16 (3.3%)	14 (1.5%)	0.03	0.094
Autoimmune Disease, Yes	17 (1.5%)	44 (2.1%)	0.259	0.389	11 (2.3%)	23 (2.5%)	0.772	0.831
Hepatitis B, Yes	95 (8.4%)	184 (8.6%)	0.817	0.88	57 (12%)	85 (9.3%)	0.15	0.262
ALT, U/L	15 (11, 21)	15 (11, 21)	0.308	0.438	15 (11, 21)	15 (11, 22)	0.947	0.949
AST, U/L	19.0 (16.0, 22.0)	18.0 (16.0, 22.0)	0.019	0.049	19.0 (17.0, 22.0)	19.0 (16.0, 22.0)	0.073	0.175
ALP, U/L	68 (58, 80)	65 (56, 75)	<0.001	<0.001	67 (58, 78)	65 (56, 75)	0.017	0.065
TP, g/L	68.8 (65.1, 72.1)	68.9 (65.3, 72.7)	0.144	0.258	68.2 (64.6, 72.1)	69.5 (65.9, 73.2)	<0.001	0.002
ALB, g/L	41.3 (39.3, 43.6)	41.8 (39.6, 44.1)	0.001	0.004	41.3 (38.8, 43.7)	41.9 (39.9, 44.2)	<0.001	0.001
GLB, g/L	27.2 (24.6, 29.5)	27.1 (24.4, 29.7)	0.677	0.758	27.3 (24.4, 29.4)	27.2 (24.9, 29.9)	0.407	0.517
TBIL, μmol/L	11.0 (8.9, 13.9)	11.1 (8.8, 14.4)	0.264	0.389	11.0 (8.7, 14.0)	11.1 (9.0, 14.3)	0.354	0.465
Ca, mmol/L	2.26 (2.20, 2.32)	2.28 (2.20, 2.33)	0.015	0.042	2.25 (2.19, 2.31)	2.28 (2.20, 2.33)	<0.001	0.001
PHOS, mmol/L	1.19 (1.08, 1.30)	1.19 (1.08, 1.29)	0.996	0.996	1.19 (1.09, 1.29)	1.19 (1.08, 1.30)	0.801	0.841
Na, mmol/L	140.00 (139.00, 141.00)	140.00 (139.00, 141.00)	<0.001	<0.001	140.00 (139.00, 142.00)	140.00 (139.00, 141.00)	0.129	0.246
K, mmol/L	3.99 (3.82, 4.17)	3.99 (3.83, 4.17)	0.437	0.564	3.98 (3.81, 4.18)	4.00 (3.85, 4.17)	0.162	0.273
Cl, mmol/L	106.00 (104.00, 107.00)	106.00 (104.00, 107.00)	0.002	0.007	106.00 (104.00, 107.00)	106.00 (104.00, 107.00)	0.189	0.305
CO2, mmol/L	26.00 (24.00, 27.00)	25.00 (24.00, 27.00)	<0.001	0.002	26.00 (24.00, 27.00)	25.00 (24.00, 27.00)	0.109	0.235
GLU, mmol/L	4.70 (4.40, 5.10)	4.70 (4.30, 5.00)	<0.001	0.002	4.70 (4.40, 5.10)	4.70 (4.40, 5.00)	0.007	0.031

UREA, mmol/L	4.50 (3.90, 5.30)	4.40 (3.70, 5.10)	<0.001	<0.001	4.50 (3.80, 5.30)	4.40 (3.60, 5.10)	0.004	0.022
CREA, µmol/L	62 (54, 73)	62 (55, 74)	0.417	0.559	61 (55, 72)	62 (54, 73)	0.657	0.735
UA, µmol/L	329 (286, 393)	331 (280, 398)	0.691	0.764	326 (273, 389)	328 (279, 393)	0.226	0.333
AG	13.00 (11.00, 14.00)	13.00 (12.00, 14.00)	0.002	0.007	13.00 (11.00, 14.00)	13.00 (12.00, 14.00)	0.094	0.207
Osm, mOsm/kg	290.0 (287.0, 293.0)	289.0 (286.0, 292.0)	<0.001	<0.001	290.0 (287.0, 293.0)	289.0 (286.0, 292.0)	0.005	0.024
CHOL, mmol/L	4.70 (4.40, 5.40)	4.70 (4.20, 5.20)	<0.001	0.003	4.70 (4.40, 5.20)	4.70 (4.20, 5.10)	0.01	0.04
TG, mmol/L	1.06 (0.87, 1.37)	1.06 (0.81, 1.37)	0.023	0.059	1.06 (0.87, 1.45)	1.06 (0.81, 1.37)	0.123	0.24
LDLc, mmol/L	2.93 (2.68, 3.39)	2.93 (2.57, 3.35)	0.015	0.042	2.93 (2.68, 3.36)	2.93 (2.57, 3.20)	0.034	0.1
HDLc, mmol/L	1.22 (1.12, 1.39)	1.22 (1.07, 1.36)	<0.001	0.003	1.22 (1.13, 1.41)	1.22 (1.07, 1.36)	0.001	0.011
ApoA1, g/L	1.28 (1.20, 1.41)	1.28 (1.17, 1.36)	<0.001	<0.001	1.28 (1.22, 1.43)	1.28 (1.15, 1.37)	<0.001	<0.001
ApoB, g/L	0.83 (0.76, 0.97)	0.83 (0.72, 0.93)	<0.001	<0.001	0.83 (0.75, 0.96)	0.83 (0.71, 0.91)	0.003	0.017
ApoE, mg/L	39 (35, 45)	39 (34, 44)	0.06	0.133	39 (36, 45)	39 (35, 44)	0.061	0.16
LPa, mg/L	98 (63, 177)	98 (58, 177)	0.142	0.258	98 (68, 194)	98 (58, 162)	0.005	0.024
WBC, $\times10^9$/L	5.99 (5.13, 7.07)	5.96 (5.07, 6.99)	0.232	0.361	5.84 (5.04, 6.89)	5.98 (5.13, 7.04)	0.15	0.262
NEUT, $\times10^9$/L	3.30 (2.70, 4.10)	3.30 (2.67, 4.03)	0.812	0.88	3.20 (2.60, 3.98)	3.32 (2.65, 4.08)	0.118	0.235
LY, $\times10^9$/L	2.00 (1.65, 2.41)	2.00 (1.65, 2.33)	0.204	0.33	1.99 (1.69, 2.35)	2.00 (1.66, 2.40)	0.607	0.699
MO, $\times10^9$/L	0.41 (0.34, 0.50)	0.41 (0.33, 0.50)	0.443	0.564	0.40 (0.32, 0.50)	0.41 (0.33, 0.50)	0.561	0.654
EO, $\times10^9$/L	0.12 (0.09, 0.20)	0.12 (0.09, 0.20)	0.985	0.996	0.13 (0.09, 0.23)	0.12 (0.09, 0.20)	0.039	0.105
BASO, $\times10^9$/L	0.03 (0.01, 0.04)	0.03 (0.01, 0.04)	0.16	0.28	0.03 (0.01, 0.05)	0.03 (0.01, 0.04)	0.269	0.39
RBC, $\times10^{12}$/L	4.56 (4.24, 4.92)	4.57 (4.26, 4.96)	0.419	0.559	4.55 (4.21, 4.91)	4.57 (4.28, 4.96)	0.217	0.331
Hb, g/L	133 (125, 143)	133 (124, 144)	0.829	0.881	132 (121, 142)	133 (124, 144)	0.027	0.09
Ht, %	0.40 (0.38, 0.43)	0.40 (0.37, 0.43)	0.991	0.996	0.40 (0.37, 0.42)	0.40 (0.37, 0.43)	0.068	0.169
MCHC, g/L	334 (327, 340)	334 (328, 341)	0.395	0.544	333 (326, 339)	334 (327, 341)	0.029	0.094
MCH, pg	29.7 (28.5, 30.8)	29.7 (28.4, 30.7)	0.481	0.598	29.5 (28.1, 30.6)	29.7 (28.4, 30.7)	0.082	0.191
MCV, fL	89 (85, 91)	88 (85, 91)	0.087	0.183	88 (84, 91)	88 (85, 91)	0.402	0.517
RDW, %	0.130 (0.120, 0.140)	0.130 (0.120, 0.130)	0.005	0.016	0.130 (0.120, 0.140)	0.130 (0.120, 0.140)	0.002	0.017

PLT, $\times 10^9$/L	235 (203, 275)	238 (203, 278)	0.222	0.352	239 (199, 278)	241 (206, 282)	0.306	0.435
PCT, %	0.23 (0.20, 0.27)	0.24 (0.20, 0.28)	0.007	0.021	0.24 (0.19, 0.28)	0.24 (0.21, 0.28)	0.027	0.09
MPV, fL	10.00 (9.12, 10.70)	10.10 (9.30, 10.80)	0.012	0.034	10.10 (9.20, 10.60)	10.10 (9.50, 10.90)	0.001	0.011
PDW, %	12.70 (11.20, 16.07)	12.70 (11.30, 16.10)	0.432	0.564	12.70 (11.20, 16.01)	12.70 (11.30, 15.97)	0.463	0.573
PT, s	11.20 (10.70, 11.70)	11.20 (10.70, 11.70)	0.306	0.438	11.20 (10.80, 11.70)	11.20 (10.70, 11.80)	0.691	0.753
PTA, %	102 (97, 108)	102 (97, 108)	0.031	0.073	102 (97, 108)	102 (97, 108)	0.931	0.949
INR	0.96 (0.91, 1.00)	0.96 (0.91, 1.00)	0.671	0.758	0.95 (0.91, 0.99)	0.96 (0.91, 1.00)	0.316	0.442
APTT, s	28.6 (26.1, 30.9)	29.1 (26.7, 31.8)	<0.001	<0.001	28.7 (26.6, 31.4)	29.2 (26.7, 32.0)	0.2	0.311
TT, s	17.70 (17.00, 18.40)	17.60 (17.00, 18.40)	0.102	0.204	17.70 (17.00, 18.40)	17.60 (16.90, 18.20)	0.022	0.081
Fbg, g/L	2.57 (2.24, 2.99)	2.47 (2.14, 2.85)	<0.001	<0.001	2.48 (2.19, 2.88)	2.49 (2.18, 2.89)	0.883	0.915
SG	1.019 (1.014, 1.023)	1.020 (1.015, 1.025)	<0.001	<0.001	1.020 (1.014, 1.024)	1.020 (1.015, 1.024)	0.222	0.332
UGE, pos	341 (30%)	565 (26%)	0.028	0.069	131 (27%)	253 (28%)	0.781	0.831
UNIT, pos	25 (2.2%)	30 (1.4%)	0.09	0.185	10 (2.1%)	10 (1.1%)	0.147	0.262
UG, pos	10 (0.9%)	15 (0.7%)	0.575	0.673	8 (1.6%)	6 (0.7%)	0.092	0.207
UP, pos	100 (8.8%)	201 (9.4%)	0.577	0.673	46 (9.5%)	73 (8.0%)	0.342	0.464
UKB, pos	56 (4.9%)	107 (5.0%)	0.929	0.963	21 (4.3%)	62 (6.8%)	0.064	0.163
URO, pos	12 (1.1%)	42 (2.0%)	0.052	0.119	6 (1.2%)	16 (1.7%)	0.461	0.573
UBG, pos	4 (0.4%)	16 (0.7%)	0.166	0.28	2 (0.4%)	9 (1.0%)	0.348	0.464
UOB, pos	333 (29%)	573 (27%)	0.123	0.234	140 (29%)	228 (25%)	0.115	0.235
UEC, pos	337 (30%)	700 (33%)	0.074	0.159	141 (29%)	330 (36%)	0.008	0.033
BACT, pos	284 (25%)	591 (28%)	0.106	0.208	110 (23%)	273 (30%)	0.004	0.022

1. Median (IQR) or Frequency (%)

2. Pearson Chi-squared test; Wilcoxon rank sum test

3. P values for group comparisons were adjusted by the false discovery rate (FDR) correction.

Supplemental Table 3. The Discrimination Ability of Different Models during Feature Screening.

AUC	LR	LASSO	RF	GBDT	XGBoost	SVM
**Models trained with all variables**						
Train set	0.78 (0.77-0.8)	0.76 (0.74-0.78)	1 (1-1)	0.95 (0.94-0.96)	0.9 (0.88-0.91)	0.8 (0.79-0.82)
Test set	0.74 (0.71-0.77)	0.74 (0.71-0.77)	0.73 (0.71-0.76)	0.75 (0.72-0.77)	0.75 (0.72-0.77)	0.62 (0.59-0.64)
**Models trained with only demographic characteristics and thyroid biomarkers**						
Train set	0.76 (0.75-0.78)	0.76 (0.74-0.78)	1 (1-1)	0.85 (0.84-0.86)	0.85 (0.84-0.86)	0.65 (0.64-0.67)
Test set	0.74 (0.72-0.77)	0.74 (0.71-0.77)	0.73 (0.7-0.76)	0.74 (0.71-0.77)	0.74 (0.71-0.76)	0.63 (0.61-0.66)

1. Model discrimination ability was assessed using the area under the receiver operating characteristic curve (AUC).

2. LR, logistic regression; RF, Random Forest; GBDT, Gradient Boosting Decision Tree; XGBoost, ExtremeGradient Boosting; SVM, Support Vector Machine.

Supplemental Table 4. Baseline characteristics of the subgroups cohort for the external validation cohort.

Variable	Overall N = 299[1]	Benign N = 117[1]	Malignant N = 182[1]	p-value[2]
**Gender**				0.687
Female	216 (72%)	83 (71%)	133 (73%)	
Male	83 (28%)	34 (29%)	49 (27%)	
**Age, y**	47 (37, 56)	53 (40, 62)	43 (34, 52)	<0.001
**FT3, pmol/L**	3.96 (3.65, 4.26)	3.97 (3.60, 4.36)	3.96 (3.68, 4.24)	0.568
**FT4, pmol/L**	12.42 (11.31, 13.52)	12.31 (11.06, 13.52)	12.45 (11.44, 13.49)	0.511
**TT3, nmol/L**	1.35 (1.24, 1.51)	1.34 (1.25, 1.55)	1.36 (1.23, 1.50)	0.489
**TT4, nmol/L**	94 (84, 104)	94 (82, 105)	94 (85, 103)	0.856
**TSH, mIU/L**	1.27 (0.76, 1.96)	1.09 (0.67, 1.59)	1.40 (0.85, 2.09)	0.002
**lnTg, ng/mL**	2.72 (2.03, 3.42)	3.26 (2.48, 4.71)	2.39 (1.68, 3.05)	<0.001
**TGAB, IU/mL**				0.324
≤40	253 (85%)	102 (87%)	151 (83%)	
>40	46 (15%)	15 (13%)	31 (17%)	
**TPOAb, IU/mL**				0.486
≤35	250 (84%)	100 (85%)	150 (82%)	
>35	49 (16%)	17 (15%)	32 (18%)	

1. Median (IQR) or Frequency (%)

2. Fisher's exact test; Wilcoxon rank sum test

Supplemental Table 5. Baseline characteristics of the ultrasound data.

Variable	Overall	Benign	Malignant	p-value[2]
	N = 165[1]	N = 69[1]	N = 96[1]	
**Age, years**	46 (37, 55)	52 (39, 62)	44 (35, 52)	0.001
**lnTg, ng/mL**	2.77 (2.03, 3.56)	3.39 (2.70, 4.85)	2.25 (1.42, 3.01)	<0.001
**TI-RADS**				<0.001
1	2 (1.2%)	1 (1.4%)	1 (1.0%)	
2	21 (13%)	20 (29%)	1 (1.0%)	
3	29 (18%)	27 (39%)	2 (2.1%)	
4	34 (21%)	16 (23%)	18 (19%)	
5	79 (48%)	5 (7.2%)	74 (77%)	
**Size, mm**	12 (7, 28)	31 (13, 48)	8 (6, 13)	<0.001

1. Median (IQR) or Frequency (%)

2. Fisher's exact test; Wilcoxon rank sum test

Supplemental Table 6. Sensitivity Analysis of Tg-age model: Adjustment for Nodule Size in the External Validation Cohort

	**Crude model**		**Adjusted model**	
	Odds Ratio (95% CI)	p-value	Odds Ratio (95% CI)	p-value
Age, years	0.95 (0.92-0.98)	< 0.001	0.94 (0.91-0.98)	0.002
lnTg, ng/mL	0.43 (0.31-0.59)	0.001	0.62 (0.45-0.86)	0.004
Size, mm	/	/	0.9 (0.86-0.94)	< 0.001

This analysis was based on the external validation cohort; among these, 165 patients had complete ultrasound data. Nodule size was defined as the maximal diameter measured by ultrasound. Data are presented as odds ratios (OR) with 95% confidence interval (CI).

Supplemental Table 7. Performance of the predictive model across different clinical subgroups (AUC and 95% confidence interval).

Dataset	Variable	Subgroup	AUC (95%CI)[1]
Training	Gender	Female	0.748 (0.728-0.769)
Training		Male	0.778 (0.745-0.81)
Internal Validation		Female	0.728 (0.696-0.76)
Internal Validation		Male	0.755 (0.703-0.807)
External Validation		Female	0.798 (0.737-0.859)
External Validation		Male	0.742 (0.635-0.85)
Training	TSH[2]	lower	0.753 (0.729-0.777)
Training		upper	0.743 (0.716-0.77)
Internal Validation		lower	0.731 (0.693-0.769)
Internal Validation		upper	0.727 (0.685-0.768)
External Validation		lower	0.792 (0.727-0.858)
External Validation		upper	0.751 (0.651-0.851)
Training	TGAB[2]	≤40	0.749 (0.731-0.768)
Training		>40	0.706 (0.625-0.786)
Internal Validation		≤40	0.724 (0.695-0.753)
Internal Validation		>40	0.798 (0.706-0.89)
External Validation		≤40	0.776 (0.718-0.835)
External Validation		>40	0.845 (0.717-0.974)
Training	TPOAb[2]	≤35	0.752 (0.733-0.771)
Training		>35	0.728 (0.676-0.779)
Internal Validation		≤35	0.722 (0.692-0.752)
Internal Validation		>35	0.779 (0.71-0.847)
External Validation		≤35	0.776 (0.717-0.835)
External Validation		>35	0.836 (0.723-0.95)

1. AUC refers to the area under the receiver operating characteristic curve.

2. TSH levels were dichotomized based on the median; TGAb and TPOAb were dichotomized based on their normal ranges.

Supplemental Table 8. List of instrumental variables for Mendelian randomization analysis.

Factor	SNP	Effect allele	Other allele	BETA	SE	p-value	Sample size	EAF
TSH	rs10748781	A	C	-0.059	0.003	1.97E-87	264864	0.574
TSH	rs17020127	A	G	-0.103	0.005	1.57E-88	265816	0.913
TSH	rs11038357	A	T	-0.065	0.003	1.26E-92	269086	0.285
TSH	rs10799824	A	G	-0.119	0.004	5.12E-194	260264	0.156
TSH	rs768356	T	C	-0.074	0.004	1.35E-92	266599	0.800
TSH	rs334699	A	G	-0.145	0.007	6.89E-98	265863	0.056
TSH	rs73575083	A	G	0.092	0.003	3.03E-199	269086	0.675
TSH	rs7248104	A	G	-0.058	0.003	3.20E-87	261188	0.406
TSH	rs737308	T	G	-0.093	0.003	1.02E-177	265703	0.276
TSH	rs13138273	A	G	0.111	0.004	1.00E-200	266845	0.799
TSH	rs2928167	A	G	0.133	0.004	1.00E-200	267872	0.865
TSH	rs1993945	A	T	-0.149	0.003	1.00E-200	267872	0.618
TSH	rs1033701	A	G	-0.117	0.003	1.00E-200	267222	0.273
TSH	rs2396083	C	G	0.097	0.003	1.00E-200	269086	0.685
TSH	rs9296422	C	G	-0.077	0.003	1.65E-117	269086	0.248
TSH	rs925489	T	C	0.075	0.003	4.50E-138	271027	0.653
FT3	rs1169288	A	C	-0.032	0.006	2.21E-07	54953	0.679
FT3	rs4149115	A	G	0.044	0.008	8.87E-08	54257	0.149
FT3	rs225015	A	G	0.033	0.006	1.60E-07	53073	0.329
FT3	rs12085757	T	C	0.030	0.006	4.49E-07	57107	0.379
FT3	rs2235544	A	C	-0.070	0.006	1.87E-34	57107	0.523
FT3	rs1275965	T	C	0.031	0.006	3.02E-07	57107	0.385
FT3	rs784742	T	C	0.066	0.013	1.59E-07	57107	0.944
FT3	rs17628883	A	G	-0.057	0.010	3.89E-09	53073	0.103
FT3	rs1521985	T	C	-0.035	0.006	2.71E-09	53073	0.485
FT3	rs78677597	A	C	-0.052	0.007	8.49E-14	57107	0.770
FT3	rs4721388	A	C	0.033	0.006	2.25E-07	57107	0.695
FT3	rs1588635	A	C	0.069	0.006	1.44E-28	53073	0.343
FT3	rs4743032	A	T	-0.036	0.007	2.47E-07	53073	0.768
FT3	rs4842131	T	C	-0.060	0.006	4.61E-22	51314	0.435
FT4	rs10838738	A	G	0.038	0.004	2.28E-17	112987	0.654
FT4	rs4762679	T	C	-0.055	0.006	5.27E-18	117166	0.134
FT4	rs11626434	C	G	0.059	0.004	4.62E-41	117166	0.356
FT4	rs1352814	T	C	0.048	0.005	2.92E-27	117166	0.681
FT4	rs2235544	A	C	0.139	0.004	1.00E-200	111048	0.525
FT4	rs8038670	T	G	0.037	0.004	5.91E-17	117166	0.358
FT4	rs4146836	C	G	-0.037	0.004	3.18E-18	117166	0.536
FT4	rs178791	A	C	0.039	0.004	1.06E-20	119107	0.468

FT4	rs16962266	T	C	-0.087	0.010	9.54E-19	117166	0.949
FT4	rs56069042	A	G	0.112	0.012	2.31E-20	111048	0.964
FT4	rs6430552	T	C	-0.041	0.004	7.63E-21	113797	0.412
FT4	rs11675434	T	C	-0.040	0.004	5.78E-20	108622	0.405
FT4	rs112649654	T	G	-0.105	0.007	7.71E-49	115211	0.902
FT4	rs17185536	T	C	0.066	0.005	3.00E-38	117166	0.238
FT4	rs75705948	A	G	0.045	0.004	3.17E-24	117166	0.638
FT4	rs9356988	A	G	-0.042	0.005	5.45E-19	117166	0.268
FT4	rs6471865	A	C	0.050	0.006	2.94E-16	117166	0.866
FT4	rs965513	A	G	-0.083	0.004	1.39E-78	112989	0.340
FT4	rs7858917	A	T	-0.038	0.005	2.14E-16	117166	0.295
FT4	rs10760344	T	G	-0.040	0.005	1.98E-19	112898	0.336
FT4	rs4842131	T	C	-0.087	0.005	1.12E-83	112708	0.439
TT3	rs1169281	A	G	0.074	0.012	8.94E-10	15829	0.323
TT3	rs10083137	A	G	-0.152	0.031	6.39E-07	15829	0.963
TT3	rs61987066	T	C	0.067	0.015	3.46E-06	15829	0.792
TT3	rs28929474	T	C	0.191	0.041	2.43E-06	15829	0.022
TT3	rs2235544	A	C	-0.055	0.011	1.25E-06	15829	0.523
TT3	rs139402934	T	C	-0.257	0.055	3.49E-06	15012	0.988
TT3	rs8060937	T	C	-0.092	0.019	7.83E-07	15829	0.886
TT3	rs116622946	C	G	-0.205	0.044	3.19E-06	15829	0.977
TT3	rs9320394	T	C	-0.052	0.011	4.86E-06	15829	0.427
TT3	rs56167634	A	G	0.125	0.027	3.59E-06	15829	0.939
TT3	rs113330983	T	C	-0.195	0.041	2.05E-06	14037	0.966
TT3	rs925489	T	C	-0.072	0.012	1.68E-09	15829	0.665
TT3	rs112301964	A	G	-0.186	0.041	4.95E-06	13192	0.962
TT4	rs12138119	T	C	-0.116	0.025	4.64E-06	3967	0.248
TT4	rs12994425	T	C	-0.293	0.066	8.33E-06	3996	0.029
TT4	rs803366	A	G	-0.119	0.027	9.54E-06	4014	0.203
TT4	rs78040246	G	A	0.331	0.068	1.17E-06	4022	0.026
TT4	rs139037649	A	G	-0.401	0.088	4.80E-06	3981	0.016
TT4	rs12156379	G	A	-0.382	0.079	1.31E-06	3996	0.019
TT4	rs76401187	A	G	0.281	0.062	6.85E-06	4022	0.032
TT4	rs12278575	A	C	0.307	0.067	4.42E-06	4021	0.026
TT4	rs73151067	T	C	0.114	0.024	2.38E-06	4015	0.279
TT4	rs78826075	C	T	-0.357	0.076	2.84E-06	3943	0.020
TT4	rs9570470	A	T	-0.189	0.042	8.64E-06	4016	0.068
TT4	rs138516014	G	A	-0.284	0.059	1.47E-06	4012	0.036
TT4	rs146843341	A	G	-0.327	0.069	2.33E-06	3982	0.025
Tg	rs113286431	C	T	0.398	0.085	2.51E-06	3301	0.026
Tg	rs403218	A	G	0.128	0.026	9.55E-07	3301	0.377
Tg	rs10797501	T	C	0.137	0.028	1.12E-06	3301	0.671
Tg	rs116408449	C	T	-0.465	0.101	4.57E-06	3301	0.016
Tg	rs111724119	G	C	0.603	0.129	3.09E-06	3301	0.011

Tg	rs10020189	T	C	0.150	0.032	2.19E-06	3301	0.265
Tg	rs115295277	G	A	0.469	0.092	3.63E-07	3301	0.018
Tg	rs10111097	A	G	0.132	0.028	3.02E-06	3301	0.281
Tg	rs111815561	G	C	-0.276	0.060	3.39E-06	3301	0.050
Tg	rs2342232	C	T	0.349	0.076	4.07E-06	3301	0.036
Tg	rs140474327	G	C	-0.492	0.093	1.15E-07	3301	0.023
Tg	rs113296305	T	C	0.349	0.075	3.39E-06	3301	0.035
TGAB	rs10889518	T	A	-0.269	0.051	0.000000127	2629	0.140
TGAB	rs58150014	G	A	0.222	0.047	0.00000239	2630	0.110
TGAB	rs13253854	C	A	0.155	0.033	0.0000033	2631	0.220
TPOAb	rs10808483	T	C	0.148	0.031	2.15E-06	16667	0.355
TPOAb	rs11602677	A	G	0.170	0.035	9.16E-07	16726	0.263
TPOAb	rs13021203	A	T	-1.304	0.282	3.75E-06	3380	0.023
TPOAb	rs16999999	T	C	0.312	0.062	4.84E-07	10250	0.088
TPOAb	rs17672919	T	C	-0.136	0.029	3.52E-06	16704	0.412
TPOAb	rs17786733	A	T	0.185	0.030	4.46E-10	16629	0.425
TPOAb	rs239935	A	G	0.129	0.028	4.47E-06	16686	0.498
TPOAb	rs2476601	A	G	0.274	0.054	4.04E-07	16724	0.094
TPOAb	rs2523567	C	G	-0.287	0.050	7.06E-09	13405	0.175
TPOAb	rs353648	T	G	0.244	0.052	2.92E-06	15739	0.116
TPOAb	rs4766517	C	G	-0.234	0.047	5.40E-07	13061	0.413
TPOAb	rs927221	A	G	-0.210	0.045	2.82E-06	16728	0.143

SNP, Single Nucleotide Polymorphism; BETA, the effect size of the effect allele on the trait; SE, Standard error of the BETA estimate; EAF, Effect Allele Frequency.

Supplemental Table 9. Summary of Mendelian Randomization analysis by multiple methods

Thyroid Nodules	Thyroid Markers	Method[1]	nSNP[2]	BETA[2]	SE[2]	p-value[2]
Benign	TSH	MRE	14	-0.174	1.226	0.890
Benign	TSH	WM	14	-0.719	0.345	0.037
Benign	TSH	IVW	14	-0.763	0.305	0.012
Benign	TSH	SM	14	-0.196	0.619	0.757
Benign	TSH	WMd	14	-0.465	0.513	0.382
Benign	FT3	MRE	11	-1.407	1.641	0.413
Benign	FT3	WM	11	-0.034	0.631	0.957
Benign	FT3	IVW	11	-0.332	0.494	0.502
Benign	FT3	SM	11	0.174	0.954	0.859
Benign	FT3	WMd	11	-0.052	0.799	0.949
Benign	FT4	MRE	17	0.369	0.681	0.596
Benign	FT4	WM	17	-0.036	0.397	0.928
Benign	FT4	IVW	17	0.126	0.308	0.682
Benign	FT4	SM	17	-0.793	0.763	0.314
Benign	FT4	WMd	17	-0.127	0.431	0.772
Benign	TT3	MRE	11	0.693	0.628	0.299
Benign	TT3	WM	11	-0.001	0.400	0.998
Benign	TT3	IVW	11	0.093	0.315	0.767
Benign	TT3	SM	11	-0.080	0.627	0.902
Benign	TT3	WMd	11	-0.141	0.562	0.807
Benign	TT4	MRE	12	-0.167	0.459	0.723
Benign	TT4	WM	12	0.107	0.273	0.694
Benign	TT4	IVW	12	0.158	0.204	0.440
Benign	TT4	SM	12	-0.119	0.519	0.823
Benign	TT4	WMd	12	-0.089	0.451	0.847
Benign	Tg	MRE	6	1.036	0.502	0.108
Benign	Tg	WM	6	0.654	0.289	0.024
Benign	Tg	IVW	6	0.622	0.227	0.006
Benign	Tg	SM	6	0.403	0.420	0.381
Benign	Tg	WMd	6	0.638	0.375	0.149
Benign	TGAB	IVW	2	-0.066	0.287	0.817
Benign	TPOAb	MRE	10	0.115	0.238	0.641
Benign	TPOAb	WM	10	0.076	0.147	0.605
Benign	TPOAb	IVW	10	0.082	0.110	0.457
Benign	TPOAb	SM	10	0.156	0.219	0.495
Benign	TPOAb	WMd	10	0.128	0.190	0.516
Malignant	TSH	MRE	11	-0.007	0.571	0.991
Malignant	TSH	WM	11	-0.373	0.218	0.088
Malignant	TSH	IVW	11	-0.195	0.165	0.236

Malignant	TSH	SM	11	-0.301	0.308	0.352
Malignant	TSH	WMd	11	-0.317	0.284	0.289
Malignant	FT3	MRE	12	0.742	1.501	0.632
Malignant	FT3	WM	12	0.218	0.437	0.617
Malignant	FT3	IVW	12	0.174	0.436	0.689
Malignant	FT3	SM	12	0.233	0.693	0.743
Malignant	FT3	WMd	12	0.280	0.498	0.585
Malignant	FT4	MRE	17	0.211	0.465	0.656
Malignant	FT4	WM	17	-0.041	0.279	0.884
Malignant	FT4	IVW	17	0.188	0.209	0.369
Malignant	FT4	SM	17	0.416	0.474	0.393
Malignant	FT4	WMd	17	0.101	0.305	0.746
Malignant	TT3	MRE	11	-0.015	0.447	0.975
Malignant	TT3	WM	11	0.073	0.293	0.803
Malignant	TT3	IVW	11	0.067	0.218	0.757
Malignant	TT3	SM	11	0.082	0.432	0.853
Malignant	TT3	WMd	11	-0.031	0.464	0.949
Malignant	TT4	MRE	9	0.553	0.433	0.242
Malignant	TT4	WM	9	0.295	0.217	0.174
Malignant	TT4	IVW	9	0.284	0.182	0.118
Malignant	TT4	SM	9	-0.182	0.420	0.677
Malignant	TT4	WMd	9	-0.128	0.357	0.730
Malignant	Tg	MRE	6	0.169	0.426	0.711
Malignant	Tg	WM	6	0.282	0.222	0.203
Malignant	Tg	IVW	6	0.230	0.175	0.188
Malignant	Tg	SM	6	0.526	0.324	0.166
Malignant	Tg	WMd	6	0.350	0.301	0.297
Malignant	TGAB	IVW	2	0.270	0.199	0.173
Malignant	TPOAb	MRE	9	0.164	0.166	0.358
Malignant	TPOAb	WM	9	0.196	0.102	0.055
Malignant	TPOAb	IVW	9	0.171	0.080	0.032
Malignant	TPOAb	SM	9	0.266	0.164	0.143
Malignant	TPOAb	WMd	9	0.234	0.146	0.149

1. Mendelian Randomization methods include inverse variance weighting (IVW), weighted median (WM), MR-Egger regression (MRE), simple median (SM), and weighted mode (WMd).

2. nSNP indicates the number of instrumental single nucleotide polymorphisms used; BETA represents the estimated causal effect; SE is the standard error; P value assesses statistical significance.

Supplemental Table 10. Correlation analysis between Tg expression and immune cell infiltration across different computational algorithms.

Infiltrates	Method	p-value	Spearman coefficients
B cell memory	CIBERSORT	0.000	-0.285
B cell memory	CIBERSORT-ABS	0.000	-0.302
B cell memory	XCELL	0.000	-0.248
B cell naive	CIBERSORT	0.001	0.156
B cell naive	CIBERSORT-ABS	0.060	0.085
B cell naive	XCELL	0.439	0.035
B cell plasma	CIBERSORT	0.000	0.395
B cell plasma	CIBERSORT-ABS	0.001	0.149
B cell plasma	XCELL	0.011	0.115
B cell	EPIC	0.044	-0.091
B cell	MCPCOUNTER	0.003	-0.134
B cell	QUANTISEQ	0.000	-0.157
B cell	TIMER	0.000	0.255
B cell	XCELL	0.000	-0.393
Class-switched memory B cell	XCELL	0.000	-0.488
Cancer associated fibroblast	EPIC	0.000	-0.496
Cancer associated fibroblast	MCPCOUNTER	0.000	-0.280
Cancer associated fibroblast	TIDE	0.001	-0.153
Cancer associated fibroblast	XCELL	0.172	-0.062
T cell CD8+ central memory	XCELL	0.024	-0.103
T cell CD8+ effector memory	XCELL	0.292	-0.048
T cell CD8+ naive	XCELL	0.000	0.184
T cell CD8+	CIBERSORT	0.117	0.071
T cell CD8+	CIBERSORT-ABS	0.000	-0.203
T cell CD8+	EPIC	0.000	0.481
T cell CD8+	MCPCOUNTER	0.000	-0.321
T cell CD8+	QUANTISEQ	0.000	-0.374
T cell CD8+	TIMER	0.000	0.257
T cell CD8+	XCELL	0.702	-0.017
T cell CD4+ (non-regulatory)	QUANTISEQ	0.000	0.457
T cell CD4+ (non-regulatory)	XCELL	0.091	-0.077
T cell CD4+ central memory	XCELL	0.000	0.409
T cell CD4+ effector memory	XCELL	0.000	-0.227
T cell CD4+ memory activated	CIBERSORT	0.007	-0.122
T cell CD4+ memory activated	CIBERSORT-ABS	0.007	-0.122
T cell CD4+ memory resting	CIBERSORT	0.000	-0.267
T cell CD4+ memory resting	CIBERSORT-ABS	0.000	-0.379
T cell CD4+ memory	XCELL	0.000	-0.263
T cell CD4+ naive	CIBERSORT	0.000	0.171

T cell CD4+ naive	CIBERSORT-ABS	0.000	0.171
T cell CD4+ naive	XCELL	0.002	-0.143
T cell CD4+ Th1	XCELL	0.216	-0.056
T cell CD4+ Th2	XCELL	0.000	-0.287
T cell CD4+	EPIC	0.011	-0.115
T cell CD4+	TIMER	0.018	-0.107
T cell regulatory (Tregs)	CIBERSORT	0.000	-0.431
T cell regulatory (Tregs)	CIBERSORT-ABS	0.000	-0.475
T cell regulatory (Tregs)	QUANTISEQ	0.000	-0.284
T cell regulatory (Tregs)	XCELL	0.000	-0.320
Neutrophil	CIBERSORT	0.853	-0.008
Neutrophil	CIBERSORT-ABS	0.648	-0.021
Neutrophil	MCPCOUNTER	0.003	0.136
Neutrophil	QUANTISEQ	0.012	0.114
Neutrophil	TIMER	0.000	-0.398
Neutrophil	XCELL	0.000	0.157
Macrophage/Monocyte	MCPCOUNTER	0.634	-0.022
Monocyte	CIBERSORT	0.282	-0.049
Monocyte	CIBERSORT-ABS	0.000	-0.250
Monocyte	MCPCOUNTER	0.634	-0.022
Monocyte	QUANTISEQ	0.082	-0.079
Monocyte	XCELL	0.000	-0.509
Macrophage M0	CIBERSORT	0.000	-0.191
Macrophage M0	CIBERSORT-ABS	0.000	-0.265
Macrophage M1	CIBERSORT	0.050	-0.089
Macrophage M1	CIBERSORT-ABS	0.000	-0.164
Macrophage M1	QUANTISEQ	0.000	-0.655
Macrophage M1	XCELL	0.000	-0.202
Macrophage M2	CIBERSORT	0.259	0.051
Macrophage M2	CIBERSORT-ABS	0.000	-0.329
Macrophage M2	QUANTISEQ	0.704	0.017
Macrophage M2	TIDE	0.000	0.520
Macrophage M2	XCELL	0.002	0.142
Macrophage	EPIC	0.000	-0.218
Macrophage	TIMER	0.000	0.410
Macrophage	XCELL	0.000	-0.304
Macrophage/Monocyte	MCPCOUNTER	0.634	-0.022
Myeloid dendritic cell activated	CIBERSORT	0.000	-0.228
Myeloid dendritic cell activated	CIBERSORT-ABS	0.000	-0.265
Myeloid dendritic cell activated	XCELL	0.000	-0.532
Myeloid dendritic cell resting	CIBERSORT	0.000	-0.376
Myeloid dendritic cell resting	CIBERSORT-ABS	0.000	-0.397
Myeloid dendritic cell	MCPCOUNTER	0.063	-0.084
Myeloid dendritic cell	QUANTISEQ	0.000	0.454

Myeloid dendritic cell	TIMER	0.000	-0.575
Myeloid dendritic cell	XCELL	0.000	-0.617
Plasmacytoid dendritic cell	XCELL	0.003	-0.134
NK cell activated	CIBERSORT	0.003	0.132
NK cell activated	CIBERSORT-ABS	0.000	-0.227
NK cell resting	CIBERSORT	0.000	0.249
NK cell resting	CIBERSORT-ABS	0.000	0.237
NK cell	EPIC	0.000	-0.262
NK cell	MCPCOUNTER	0.002	0.137
NK cell	QUANTISEQ	0.000	-0.306
NK cell	XCELL	0.264	-0.051
Mast cell activated	CIBERSORT	0.035	-0.095
Mast cell activated	CIBERSORT-ABS	0.000	-0.192
Mast cell resting	CIBERSORT	0.001	0.152
Mast cell resting	CIBERSORT-ABS	0.004	0.131
Mast cell	XCELL	0.000	-0.191
Common lymphoid progenitor	XCELL	0.256	-0.052
Common myeloid progenitor	XCELL	0.897	0.006
Endothelial cell	EPIC	0.000	0.690
Endothelial cell	MCPCOUNTER	0.000	0.777
Endothelial cell	XCELL	0.000	0.608
Eosinophil	CIBERSORT	0.009	0.119
Eosinophil	CIBERSORT-ABS	0.009	0.119
Eosinophil	XCELL	0.059	0.086
Granulocyte-monocyte progenitor	XCELL	0.192	-0.059
Hematopoietic stem cell	XCELL	0.000	0.462
T cell follicular helper	CIBERSORT	0.074	0.081
T cell follicular helper	CIBERSORT-ABS	0.000	-0.170
T cell gamma delta	CIBERSORT	0.218	-0.056
T cell gamma delta	CIBERSORT-ABS	0.217	-0.056
T cell gamma delta	XCELL	0.010	-0.117
T cell NK	XCELL	0.000	-0.536
MDSC	TIDE	0.001	0.146
